# Exploring the oral‐gut microbiota during thyroid cancer: Factors affecting the thyroid functions and cancer development

**DOI:** 10.1002/fsn3.3538

**Published:** 2023-07-08

**Authors:** Yao Kun, Wei Xiaodong, Wang Haijun, Nie Xiazi, Qiang Dai

**Affiliations:** ^1^ Department of Nuclear Medicine Gansu Provincial Hospital Lanzhou China; ^2^ Emergency Department of Gansu Provincial Hospital Lanzhou China; ^3^ Department of Gynecology Gansu Provincial Hospital Lanzhou China; ^4^ Department of Respiratory Gansu Provincial Hospital Lanzhou China

**Keywords:** factors, gut microbiota, oral microbiota, probiotics, thyroid cancer

## Abstract

Thyroid cancer (TC) is categorized into papillary, follicular, medullary, and anaplastic. The TC is increasing in several countries, including China, the United States, the United Kingdom, Canada, France, Australia, Germany, Japan, Spain, and Italy. Thus, this review comprehensively covers the factors that affect thyroid gland function, TC types, risk factors, and symptoms. Lifestyle factors (such as nutrient consumption and smoking) and pollutants (such as chemicals and heavy metals) increased the thyroid‐stimulating hormone (TSH) levels which are directly related to TC prevalence. The conventional and recent TC treatments are also highlighted. The role of the oral and gut microbiota as well as the application of probiotics on TC are also discussed. The variations in the composition of oral and gut microbes influence the thyroid function indirectly through alteration in metabolites (such as short‐chain fatty acids) that are eminent for cellular energy metabolism. Maintenance of healthy gut and oral microbiota can help in regulating thyroid function by regulating iodine uptake. Oral or gut microbial dysbiosis can be considered as an early diagnosis factor or TC marker. High TSH during TC can increase the oral microbial diversity while disrupting the high ratio of Firmicutes and Bacteroidetes in the gut. Supplementation of probiotics as an adjuvant in TC treatment is beneficial. However, needs more extensive research to explore the direct effect of probiotics on thyroid function.

## Graphical abstract

Thyroid cancer (TC) is the utmost communal endocrine malignancy. Dietary changes can reduce the frequency of papillary, follicular, and medullary TC. Probiotics increase the *Lactobacilli* count and nutrient uptake for thyroid function.

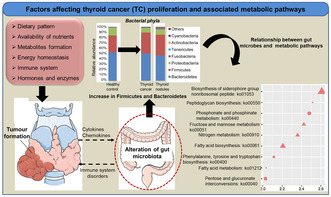

## 1 INTRODUCTION

### 1.1 Thyroid gland

**FIGURE 1:**
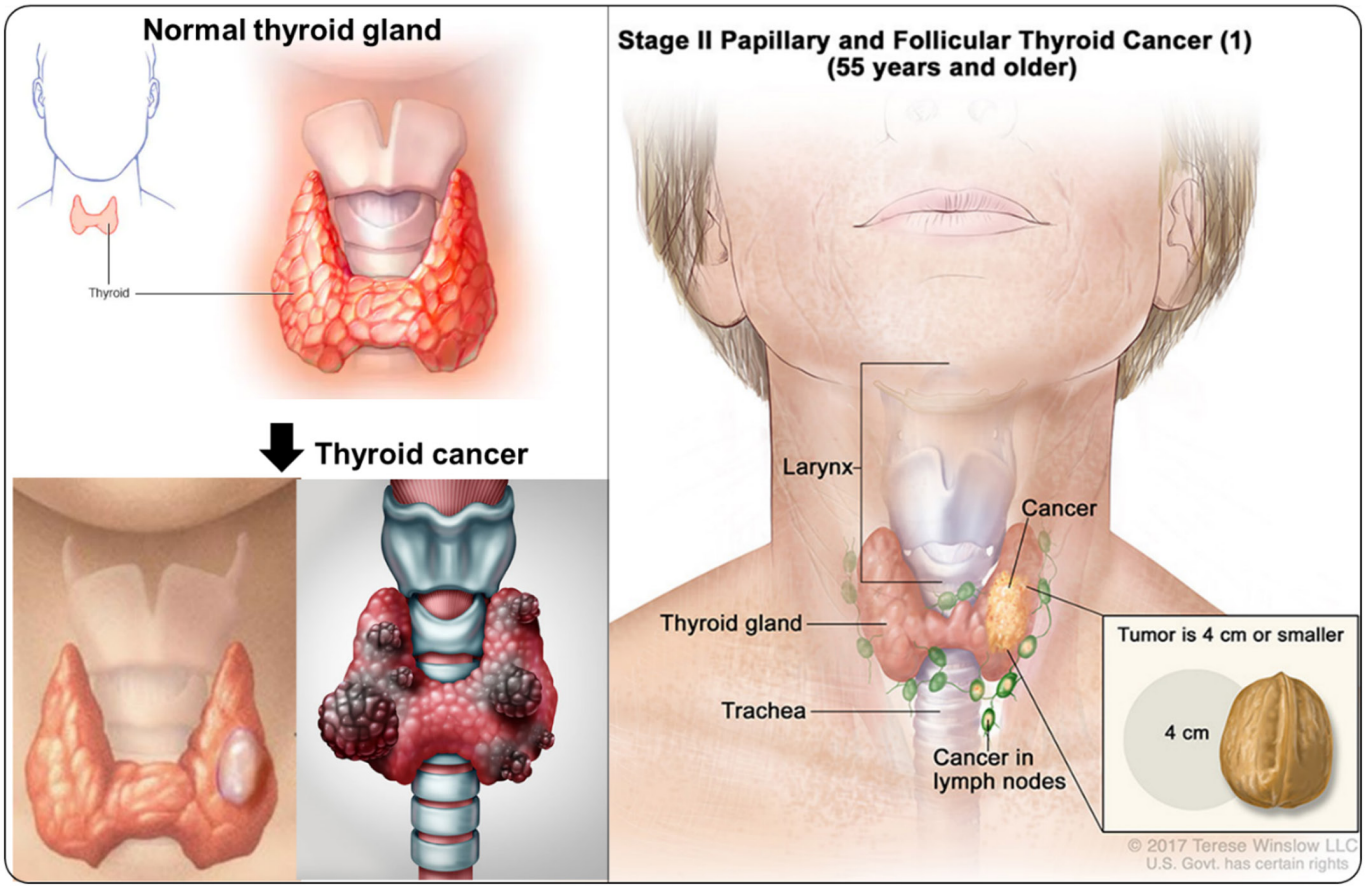
Diagrammatic representation of normal thyroid gland and thyroid cancer.

The thyroid gland (TG) is an organ located in the anterior of the neck under the thyroid cartilage and has an internal secretory role. The TG is designed like a butterfly, comprising of two lobes (on the right and the left) and the canal (called isthmus) that joins them (Figure 1). The weight of human TG differs from 15 to 20 g (Ali & Majeed, 2022). The superior thyroid, inferior thyroid, and thyroidea ima are the main central arteries that drive blood (about 5 mL) to TG. The TG is vital for cellular activities and regulation of thyroid‐stimulating hormone (TSH) by the pituitary gland. TSH is mainly involved in the synthesis of other hormones, regulating the metabolism, and growth of cells. The TG secretes hormones including triiodothyronine (T3), thyroxine (T4 or tetraiodothyronine), and calcitonin. The T3 and T4 synthesis majorly rely on iodine intake and is controlled by TSH (Gordon, 2012).

### 1.2 Thyroid diseases

Hyperthyroidism, Grave's disease, hypothyroidism, goiter, and Hashimoto's thyroiditis are the main diseases occurred during the dysfunction of TG. The history of thyroid disease in medicine started in 2700 BC through the treatment of distended thyroids in Chinese medicine by sponges and burnt seaweed containing iodine. Abul Kasim, in 1961, termed the 1st thyroid biopsy and thyroidectomy for goiter. Thomas Wharton represented the novel surgical sketches of the procedure of thyroidectomy, and coined the term “thyroid” following the Greek word “thyreos,” in 1656, based on the shape of thyroid cartilage (Werner et al., 2005). Theodor Kocher won the Nobel Prize in 1909 in “Medicine” for exploring a decrease in thyroidectomy death from 14% to 18% in 1884 and 1898, respectively (Gordon, 2012). A hospital database investigation revealed that in 2009, a total of 59,478 patients (including 30.8% malignant neoplasm and 74.8% females) were admitted who undergoes thyroidectomies (Vashishta et al., 2012). Approximately, one in 10 Americans suffers from irregular TSH levels (Canaris et al., 2000).

Hypothyroidism is a common thyroid disorder caused due to less thyroid hormone levels. It is more prevalent in women (1%–2%) compared to men (0.1%). Subclinical hypothyroidism with elevated TSH level and normal T4 count has been reported to progress to overt hypothyroidism in 5%–18% of individuals annually in the United States (McDermott & Ridgway, 2001). Thyroid disease, along with TC, is highly increasing yearly resulting in exhaustion, depression, and irregular appetite in almost 70 million Americans. The following sections report the discussion on the factors that affect thyroid function.

## 2 FACTORS THAT AFFECT THYROID FUNCTION

### 2.1 Lifestyle factors

#### 2.1.1 Nourishment

Diet components (such as soy, brassica vegetables, coffee, tea, and junk food) can alter thyroid hormones and TSH levels. The nutrients (including vitamins, trace minerals, and micro‐minerals) also affect the thyroid hormone level. The change in TSH level is due to cyanogenic glucosides and flavonoids found in various plant‐based food (de Souza Dos Santos et al., 2011; Roman, 2007). Soy‐based food comprises goitrogenic compounds known as soy isoflavones that hinder thyroid peroxidase (TPO) (Divi et al., 1997). A meta‐analysis study revealed that soya products do not affect thyroid hormone levels but rather discreetly elevate the level of TSH levels (Otun et al., 2019). The consumption of soy isoflavones also resulted in increased TSH levels (de Souza Dos Santos et al., 2011). Soy isoflavones negatively influenced the patients with subclinical hypothyroidism and pregnancy with iodine shortage (Otun et al., 2019).

Iodine (I) is an essential element for thyroid hormone synthesis, which is taken mainly from food and water. Uneven distribution of I on Earth leads to insufficient I‐intake, which has different health consequences for different age groups (Milanesi & Brent, 2017). The I transport is a rate‐defining phase during the synthesis of thyroid hormones. I deficiency can cause goiters in people of all ages, as well as an increased sensitivity of the thyroid to nuclear radiation. Hypothyroidism occurs in people with severe I deficiency. In adults, I deficiency can decrease thyroxine, and even toxic nodular goiter and hyperthyroidism can occur (Zimmermann & Boelaert, 2015). Salt iodization is the most effective way to control I deficiency (Su et al., 2018). Long‐term excessive intake of I can cause thyroid autoregulation disorders, hypothyroidism, and the risk of autoimmune diseases increases. Excess I can also increase the risk of hyperthyroidism or TC (Koukkou et al., 2017).

**FIGURE 2:**
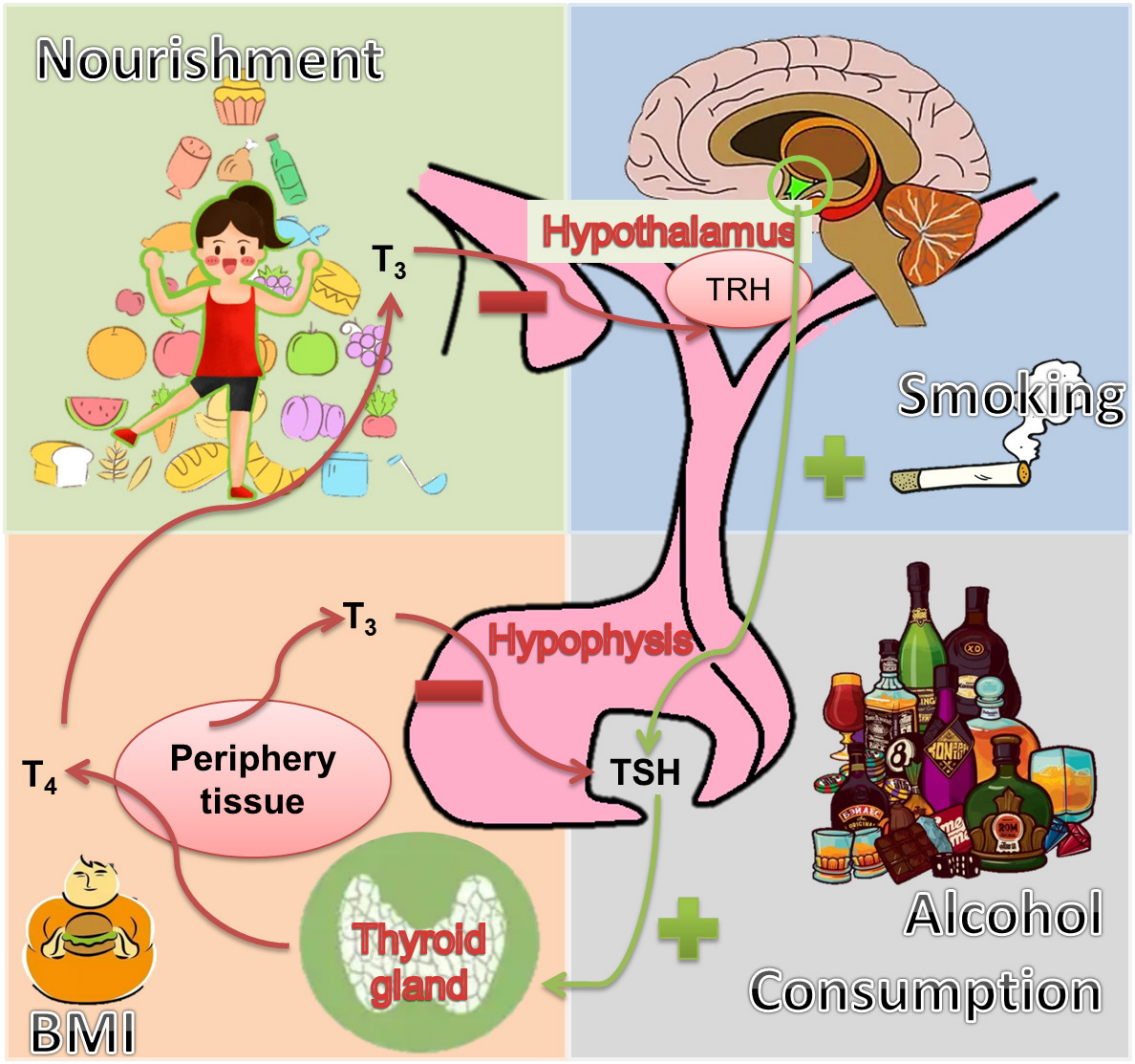
The effect of lifestyle factors (such as nourishment, smoking, alcohol consumption, and body mass index) on thyroid functioning.

The selenium (Se) intake from various sources (such as meat, seafood, and grains) also affects thyroid functioning. It helps in the biosynthesis of Selenocysteine‐containing selenoproteins which activate thyroid hormones (Winther et al., 2020). Several selenium‐containing enzymes (such as selenophosphate synthetase, Se‐containing glutathione peroxidases, thioredoxin reductase, and iodothyronine deiodinases) participate in the antioxidant network (Triggiani et al., 2009). These enzymes are abundantly present in the TG and help thyroid hormone functioning. A children's study in Morocco found that the average concentration of Se in children with goiter was low and an increase in thyroid volume was associated with Se deficiency (El‐Fadeli et al., 2016). Low Se was also linked to the risk of goiter and multiple nodules in European women (Schomburg, 2012). Other trace elements related to anti‐oxidation or thyroid function (such as Cu, Fe, Zn, Mn, Mg, and K) have also been reported (Jain, 2014; Triggiani et al., 2009). Studies on vitamin A and D deficiency and thyroid diseases have also appeared (Barrea et al., 2021). Although they are known to be essential and vital for human health, there is limited evidence that these substances are associated with thyroid disease. The effect of a single element on the TG is difficult to investigate, so there is still controversy and doubt (Figure 2).

#### 2.1.2 Cigarette smoking

Smoking is harmful to human health and the environment. Tobacco smoke produces chemicals that affect the TG (Figure 2). An epidemiological study found that smoking affected FT4 and TSH levels (Mouhamed et al., 2012). Heavy smoking increased the occurrence of multiple thyroid nodules (TN) and goiter (Aydin et al., 2011). The level of cotinine (the main metabolite of nicotine) in urine was also found to have a significant dose‐dependent effect on thyroid function and thyroid autoimmunity (Kim, Kim, et al., 2019). Moreover, smoking also affects the growth and development of the fetus (Bednarczuk et al., 2020). Maternal smoking during the second trimester affected fetal thyroid development and endocrine dysfunction (Filis et al., 2018).

Cigarette smoking reduced the TSH levels and increased fT3 and fT4 count (Gruppen et al., 2020). A study of 4249 individuals enclosed that each 10 ng/mL rise in serum cotinine decreased the TSH by 1.4% (Kim, Kim, et al., 2019). A gradual rise in TSH levels was also reported after smoking cessation (Zhang, Shi, et al., 2019). The mechanism associated with cigarette smoking that influences the levels of thyroid hormones and TSH is still unclear due to the >4000 components in tobacco. It has been concluded that the reduction of serum TSH concentration in most smokers is related to the rise of fT4. Thiocyanate (transmuted from cyanide in tobacco) hinders I transport and incorporation into Tg which reduces the synthesis of thyroid hormone. Thiocyanate reduced the T4 levels and subsequently increased the fT4 count. Smoking decreases autoimmune processes in the TG, resulting in changes in thyroid hormone levels and TSH (Wiersinga, 2013). Smoking also elevated the activity of sympathetic nervous system, which increased the level of thyroid hormone subsequently resulting in reduced TSH (Filis et al., 2018).

#### 2.1.3 Alcohol consumption

Alcohol drinking influences the thyroid hormones by raising TSH and reducing fT3 levels (Figure 2). While, no change in TSH levels and reduction in thyroid hormones was also reported (Valeix et al., 2008). Alcoholic cirrhosis increased serum Tg levels (Aoun et al., 2015). Long‐term administration of ethanol decreased the TSH through the decrease in pituitary TRH receptors (Hermann et al., 2002). Alcohol dysfunctions the thyroid by increasing the thyroid hormones and reducing the T3 count (Balhara & Deb, 2013). Furthermore, resveratrol showed thyroid disruption in vitro/in vivo (Oliveira et al., 2018).

#### 2.1.4 Body mass index

Body mass index (BMI) has a positive correlation with thyroid hormone, TSH, and fT3 levels (Dai et al., 2020; Song et al., 2019; Taylor et al., 2016). High maternal BMI improved the levels of fetal TSH and thyroid mass (Filis et al., 2018). The results of studies exploring the link between BMI and fT4 were inconsistent. The majority of the studies did not report any association between BMI and fT4, while a few studies reported either negative or positive link among BMI and fT4 (Habib et al., 2020; Xu et al., 2019). Correlation between level of thyroid hormones and weight is explored in autoimmune disorders such as hyperthyroidism (related to weight loss) and hypothyroidism (associated with an increase in weight) (Sanyal & Raychaudhuri, 2016). The secretion of leptin hormone by adipose tissue plays a major role in the synthesis of hypothalamic TRH (Paul et al., 2011). In obesity, the changes in TSH and thyroid hormone are due to the process of alteration to subclinical hypothyroidism or weight increase. However, the major factors responsible for varying TH and TSH in euthyroid persons during high BMI are not comprehended yet (Figure 2).

### 2.2 Pollutants

#### 2.2.1 Chemicals

Advances and developments in technology have been followed by various toxic chemicals or pollutants that unescapably enter the human body through air, water, food, and skin, leading to chronic diseases in human tissues. The well‐known endocrine disruptors have been found to be widely present in the blood and urine of the human body. Similar hormones act on organisms and interfere with many aspects of the endocrine system, resulting in various health abnormalities (Table 1). Chemical pollutants including a variety of organochlorine pollutants (such as polychlorinated biphenyls, also with pesticides, herbicides, and other pesticide pollutants) have gradually been found to have harmful effects on the human body.

**TABLE 1:** Summary of different pollutants and their sources that affects thyroid function.

Chemicals	Source or use	Pollution type	Harmfulness	Reference
Polychlorinated biphenyls (PCBs)	Lubrication materials, plasticizers, fungicides, heat carriers, transformer oil	With industrial wastewater and urban sewage in the water Bioaccumulation of aquatic organisms by uptake into the food chain	Interfere thyroxine production, transport, and metabolism Bind to thyroid receptors to produce agonists Harmful to the growth and development of infants Related to adult goiter and thyroid autoimmunity	Duntas and Stathatos (2015)
Bisphenol A (BPA)	Raw material for polymer synthetic materials, anti‐aging agents, plasticizers, pesticide fungicides	Use containers and plastic products containing bisphenol A, with food or water into the body Through skin and respiratory contact	Disturb the human metabolic system Prenatal exposure can decrease neonatal TSH levels BPA exposure in urine is associated with TSH levels	Duntas and Stathatos (2015)
Per and poly fluoroalkyl substances (PFAS)	Common products comprise textile coatings, non‐stick cookware coatings, food containers, personal care products, “anti‐wrinkle” and “waterproof” products, and Class B firefighting foams	Contaminated drinking water and food Use of personal care products and cookware	Reduced thyroid cell viability and proliferation rate Increased blood exposure levels associated with TSH and T4 levels. Maternal exposure during pregnancy adversely affects the infants, altering neonatal thyroid hormone levels	Rickard et al. (2022)
Pesticides	Alachlor, Dicamba, DDT, DDE, Fipronil.	User's skin, mouth, and nose direct exposure	Increase the risk of hypothyroidism in exposed persons Destroy thyroid axis and affect thyroid hormone level Some insecticides increased the incidence rate of thyroid tumors	Duntas and Stathatos (2015)
Salts of perchlorate (ClO^4−^)	Solid propellants, munitions, commercial explosives, pyrotechnics, chemical industry	Drinking water, beverages, and foods pollution	Competitive inhibition of normal iodine uptake by the human thyroid Hypothyroidism with decreased T3 and T4	Parker (2009)

#### 2.2.2 Heavy metals

Metal ions have the tendency to accumulate inside the TG and disrupt homeostasis. Some metal ions (such as Se and Zn) are essential for thyroid function, while others such as (As, Mg, Pb, and Cd) have disruptive effects (Vigneri et al., 2017). Exposure of Cd to *Rana zhenhaiensis* decreased the size and epithelial thickness of thyroid follicles (Teng et al., 2022). Subacute exposure of Pb to mice impaired thyroid function by altering the protein expression of NIS and TSHr (de Lima Junior et al., 2021). A meta‐analyses study investigated the link between metal ions and TC. Increased levels of Cu and decreased Se and Mg were observed in TC patients compared to healthy controls (Shen et al., 2015). A significant decrease was also reported in TC individuals as compared to healthy controls (Gumulec et al., 2014). These meta‐analyses suggested that the heavy metal concentration influences the cancer development in TG and needs more in‐deep research to unveil the underlying mechanism.

## 3 THYROID CANCER

Thyroid cancer (TC) is a malignant disorder of endocrine organs. The prevalence of TC has been increased in recent year, compared to other tumors. The seventh overall among the health‐threatening malignant tumors is TC in China. Follicular and parafollicular cells of thyroid can give rise to differentiated or anaplastic TC. TG carcinoma is uncommon, but nonetheless among the common malignancy (90% of all endocrine cancers).

### 3.1 TC types

TC can be categorized into four sorts based on the derivation of cells and the rate of cancer cell division as papillary TC, follicular TC, medullary TC, and anaplastic TC.

#### 3.1.1 Papillary TC

The most communal type of TC (70%–80% of TC) is papillary TC that can arise mostly between the ages of 30–60. The prevalence of papillary TC is three times higher in women compared to men and is more violent at old age. Papillary TC might spread more in the lymph nodes of neck and lesser in the lungs. Papillary TC can be healed if diagnosed early.

#### 3.1.2 Follicular TC

Follicular TC accounts for ~15% of all TCs. Hürthle cells are variations of follicular TC. Follicular TC occurs in adults, especially women between the ages of 40 and 60. Papillary TC and follicular TC are classified as distinguished thyroid carcinoma that is initiated from follicular epithelial thyroid cells. The development of follicular TC is slow and regularly having a good prediction (particularly if diagnosed early).

#### 3.1.3 Medullary TC

The medullary TC is approximately 3% of other TCs which is developed by C cells or parafollicular cells. These cells produce calcitonin to control blood Ca^2+^ and PO_4_^3−^ levels. A rise in calcitonin is an indicator of cancer. Generally, medullary TC is hereditary and more frequent in 40–50 years old men and women.

#### 3.1.4 Anaplastic TC

Anaplastic TC is infrequent and accounts for less than 2% of all TCs (77% of women). Anaplastic TC initiates from follicular cells without any innovative biological characteristics. It has rapid growth, malignancy, and high invasiveness. Anaplastic TC is more common in women above 65 years old than men. The prognosis of anaplastic TC is less and insensitive to conservative treatment. The 5‐year survival rate with anaplastic TC is almost 5%. It initiates with the immune system and also consists of thyroid lymphoma.

### 3.2 TC risk factors

#### 3.2.1 Age and gender

TC is three times more public in women than in men. TC, which can be realized at any age, happens in women at the age of 40–50 years, whereas in men at the age of 60–70 years (LeClair et al., 2021; Shrestha et al., 2023; Yao et al., 2011).

#### 3.2.2 Lacking level of iodine

Follicular TC is common in people with iodine deficiency. Foods comprising iodized salt and iodine (such as salt‐water fish, tuna, haddock, shrimp, and shellfish) ingesting can be used to remove iodine lack (Gharib, 2018).

#### 3.2.3 Radiation disclosure

Radiation exposure has been demonstrated to be a risk factor for TC. Nuclear power plants or nuclear weapons are vital sources of radiation. In the past, after the Chernobyl nuclear power plant accident in Russia, people living in that region have seen a large increase in TC. Radiation therapy applied to the head and neck area in childhood is another risk factor for TC (Iglesias et al., 2017). Risk differs according to how much radiation is given and the age of the child getting the treatment. High doses of radiation can increase the risk for children under age. If this type of treatment has been applied to you in the past, then you should definitely consult your doctor and ask for an examination of the TG. The risk of TC in adults exposed to radiation is not as high as in children (Cherella & Wassner, 2023). However, if you are exposed to radiation where you work because of the work you do, or if there is a nuclear or power plant in the area where you live, it is important to have a routine doctor's check.

#### 3.2.4 Genetic aspects

The inherited disorders are related to the development of different kinds of TC. About one in three cases of medullary TC has abnormal genes associated with a hereditary disorder. In such cases, cancer is called familial transitional medullary TC. A combination of familial transitional medullar TC and tumors that develop in other endocrine glands is termed a multipl (multiple) endocrine tumor type 2 (MEN2). Men2 has two subspecies: MEN2a and MEN2b (Accardo et al., 2017). It happens as a result of a mutation in the gene named rejection in both subspecies. MEN2a is a medullary TC that occurs with pheochromocytomas (adrenaline‐forming tumors) and parathyroid gland tumors (Moline & Eng, 2011). MEN2b is a modularized TC connected with benign tumors that develop in the tongue and nerve tissues elsewhere in the body, again named pheochromocytomas and neuroma. Moreover, hereditary diseases (such as Gardner syndrome, Cowden's disease, and familial adenomatous polyposis) also act as risk factors for TC (Punatar et al., 2012; Xu et al., 2023).

### 3.3 TC symptoms

The swelling in the neck or a fast‐growing lump, pain in the front of the neck, sometimes from the neck to the ears, hoarseness, difficulty swallowing, persistent cough, and shortness of breath are the common symptoms of TC. These symptoms are seen to play a factoring role in the early detection of TCs (Alhashemi et al., 2019). Nevertheless, it is not true that these symptoms are directly associated with TC. Other non‐serious health problems can also lead to such symptoms. In this case, it is up to you to consult a specialist doctor without wasting time. In this way, it will be possible to find a quick solution to the health problems detected early.

## 4 MICROBIOTA AND TC

Microbiota is an important factor in determining the health and disease factors. Theory of microecology stated that ecological balance between neural, endocrine, microbial, metabolic, and immune systems is eminent of life sustainability (Du et al., 2019). Oral cavity and gut are two vital gastrointestinal tract structures that comprise a microbial consortium which is mainly responsible for energy intake and metabolic processing essential for human health (Maki et al., 2021).

### 4.1 Oral microbiota and TC

**FIGURE 3:**
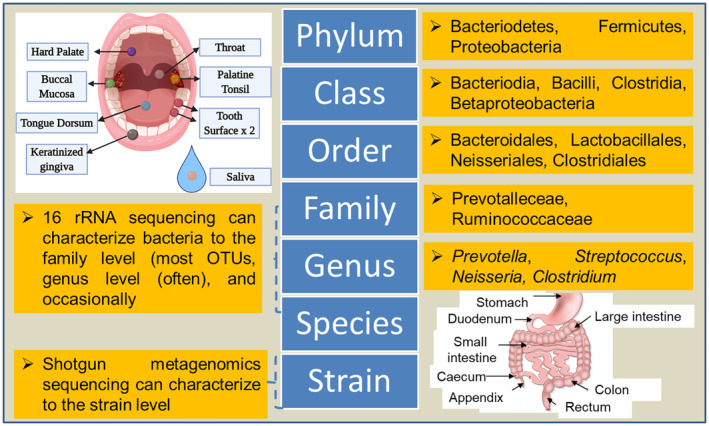
Major bacterial community of oral and gut microbiota revealed through 16S rRNA sequencing.

The oral cavity comprises different microbes which are either beneficial or related to local or systemic diseases. However, the oral microbial diversity has not been extensively studied (Figure 3). Oral microbiota has been reported to be different cellular mechanisms (such as apoptosis inhibition, high cell invasion, and increased cellular proliferation) that can directly or indirectly promote cancer formation (Tuominen & Rautava, 2021). Oral microbiota changes with thyroid‐stimulating hormone levels (TSH), as an increase in TSH, raised the taxa diversity (Dong et al., 2021). The dominance of the genus *Alloprevotella*, *Anaeroglobus*, and *Acinetobacter* was observed in salivary samples of TC patients. While, *C. Saccharibacteria* bacterium, unclassified *Clostridiales* bacterium, *Mobiluncus*, *Treponema*, unclassified Prevotellaceae, and *Acholeplasma* were highly prevalent in thyroid nodules patients (Jiao et al., 2022). The transition of microbes from oral to gut and vice‐versa can influence thyroid diseases and play a major role in TC development. However, studies on the interaction between oral microbiota and TC development are rare to conclude any underlying mechanism and need more metagenomic‐based research.

### 4.2 Gut microbiota and TC

**FIGURE 4:**
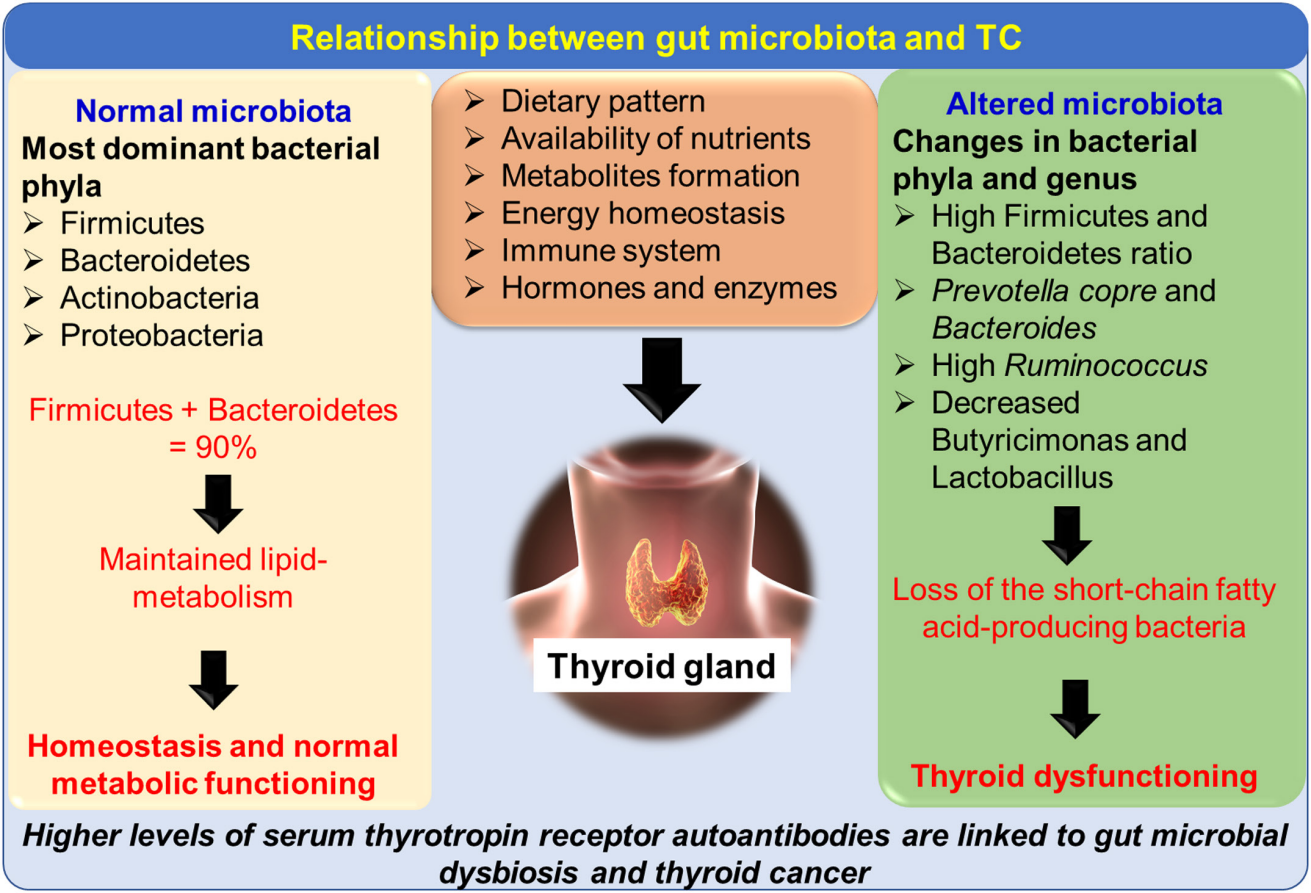
The influence of different gut microbes on thyroid gland function and complications associated with TC.

Gut microbiota consists of almost 1200 bacterial species along with bacteriophages, fungal species, and viruses. Major bacteria are Firmicutes, Bacterioidetes, Proteobacteria, Actinobacteria, and Verrucomicrobia (Figure 4). These bacteria are crucial for digestive equilibrium, immunology, hormonal balance, and metabolic homeostasis (Rinninella et al., 2019). Disruption of gut microbiota composition causes imbalance in the microbial ecosystem and a reduction in microbial diversity which is defined as dysbiosis (Rinninella et al., 2019). Metabolic and inflammatory disorders (diabetes, autoimmune diseases, inflammatory bowel disease) are mostly associated with changes in the gut microbial count (Afzaal et al., 2022). The complexity of gut microbial composition increases with age. Whereas, prolonged dietary changes or drug intake can also alter the gut microbes in adults.

The relationship between gut microbes and thyroid function has been reported in many studies (Hou et al., 2023; Knezevic et al., 2020; Zhang, Zhang, et al., 2019). The homeostasis of the intestinal epithelium is controlled by T3 through its interactions with TRα1, the dominant TR isoform in the intestine. This homeostasis depends on tight regulation of local T3 concentrations, regulated by specific TH transporters and deiodination enzymes in the intestine (Fenneman & Bruinstroop, 2023). A balanced gut microbiome is essential for the maintenance of immune and endocrine systems. Gut microbiota is also linked with Hashimoto's thyroiditis (Virili et al., 2018; Zhang, Zhang, et al., 2019), thyroid carcinoma (Yu et al., 2022), Graves' disease (Hou et al., 2021), and primary hypothyroidism (Su et al., 2020). Gut microbes affect the immune system by several metabolites which are also involved in the regulation of thyroid function (Knezevic et al., 2020). Several inflammatory disorders can contribute to microbial dysbiosis (Rinninella et al., 2019; Virili et al., 2018; Yu et al., 2022).

The most prevalent thyroid disorder is TC and thyroid nodules (TN). Thyroid metabolism can be altered by the availability of nutrients, hormones, and the functioning of gut microbes. Gut microbiota regulates the thyroid metabolism indirectly and can influence the diagnosis and treatment of cancer and melanoma (Knezevic et al., 2020; Li et al., 2021). Dietary choices affect the composition of gut microbiota and could indirectly influence thyroid functioning leading to several complications (Dong et al., 2022). Implications of gut microbiota in TC and its effect on metabolic pathways have been reported (Figure 4). These microbes affect thyroid hormonal balance by regulating iodine uptake and enterohepatic cycling (Fröhlich & Wahl, 2019). Interaction between gut microbes and thyroid hormones also includes changes in bile acids and lipid metabolism which can enhance TC. Gut‐associated microbes produce short‐chain fatty acids for enterocyte's energy metabolism resulting in strengthened enterocyte differentiation (Kunc et al., 2016). Gut microbiota influences the uptake of iodine, selenium, and iron, and the microbiota may alter the availability of l‐thyroxine (Fröhlich & Wahl, 2019). The most dominant bacterial phyla present in a healthy gut are Firmicutes, Bacteroidetes, Actinobacteria, and Proteobacteria (Fernández‐García et al., 2021).

**FIGURE 5:**
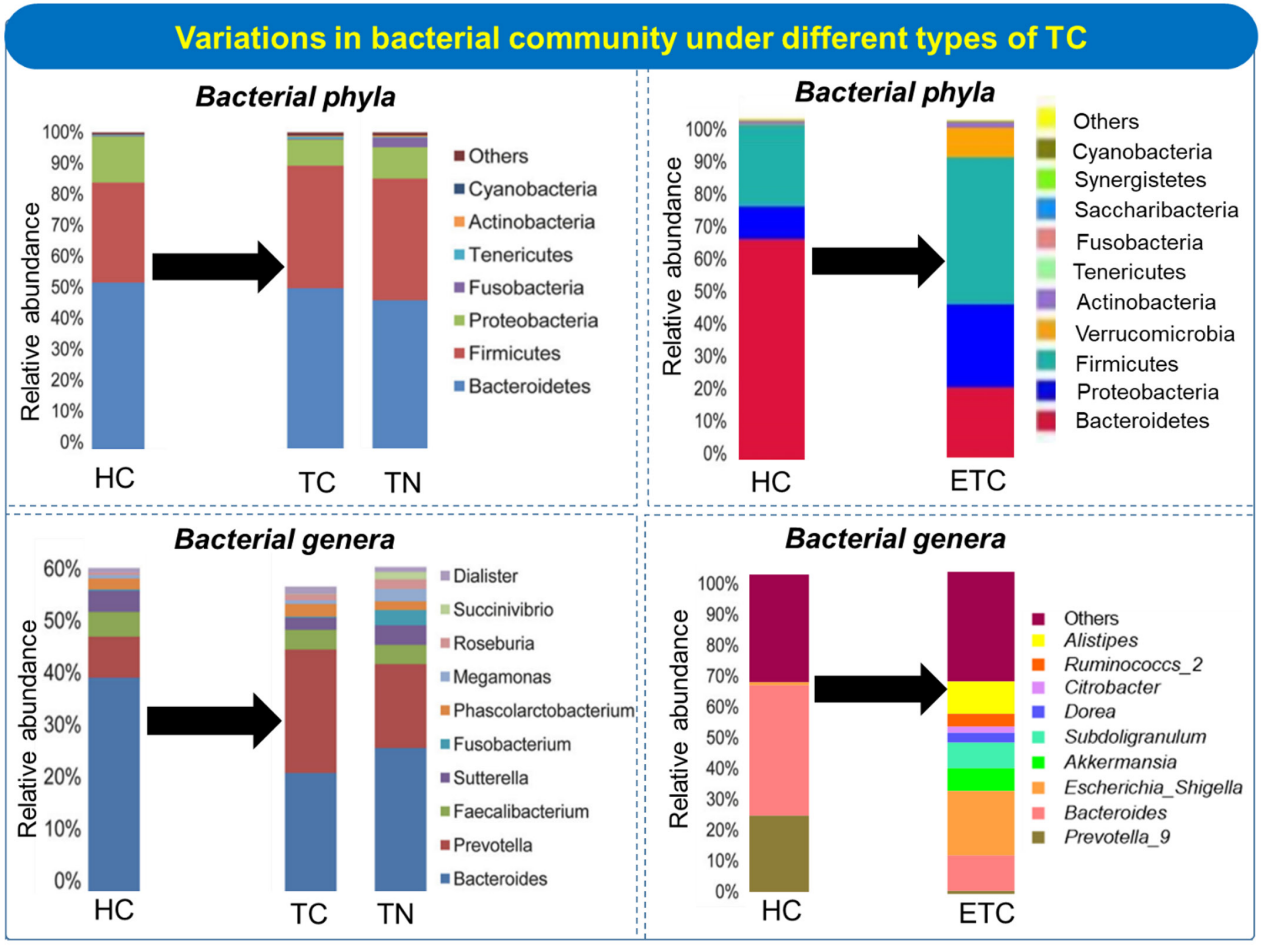
Bacterial community shifts at phylum and genus level among healthy controls (HC), thyroid cancer (TC), thyroid nodule (TN), and euthyroid thyroid cancer (ETC) patients (Ishaq et al., 2022; Zhang, Zhang, et al., 2019).

**FIGURE 6:**
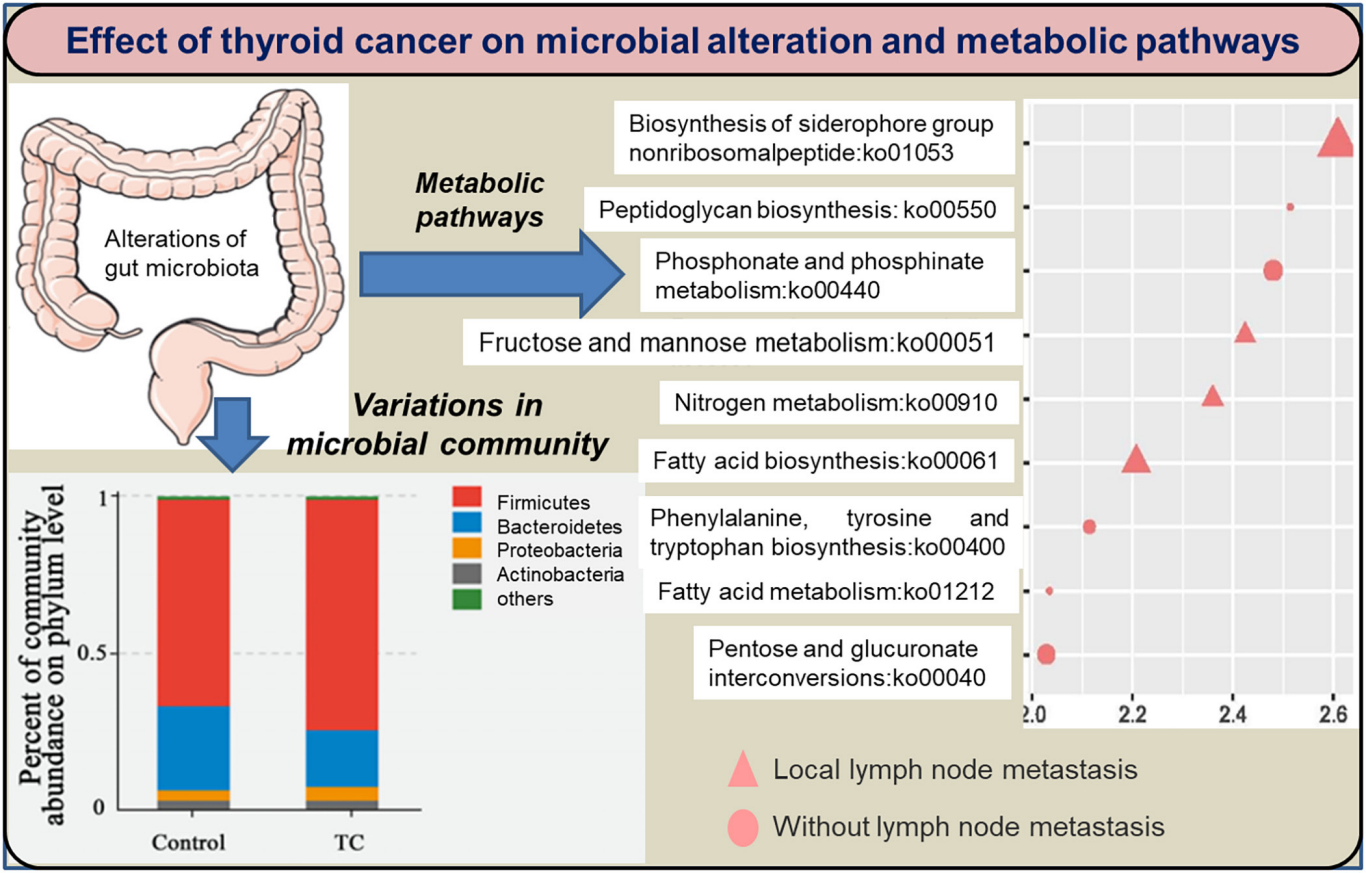
Effect of gut dysbiosis on cell proliferation‐associated metabolic pathways (Yu et al., 2022).

Gut microbial alteration or microbial dysbiosis has been studied in TC patients, indicating their indirect role in TC proliferation (Table 2). Gut microbiome analysis of 74 TC patients showed that gut microbial alteration is related to both TC and thyroid nodules. *Neisseria* and *Streptococcus*'s relative abundance was significantly higher compared to healthy individuals in both TC and thyroid nodules patients (Figure 5). A high count of *Streptococcus* has been reported to increase the menace of adenomas and carcinomas, while *Neisseria* has been related to inflammatory disorders. A notable decrease was reported for *Butyricimonas* and *Lactobacillus* for TC and thyroid nodules, respectively (Zhang, Zhang, et al., 2019). *Lactobacillus* is involved in selenium metabolism and possesses antioxidative effects on the thyroid gland. An integrated LC–MS‐based metabolomics approach revealed that the *Christensenellaceae*_R‐7 and *Eubacterium*_*coprostanoligenes* genera along with 27‐hydroxycholesterol (27HC) and cholesterol metabolites (involved in lipid metabolism) reduced in TC groups (Lu et al., 2022). A decrease in richness and diversity of gut microbes resulted in the loss of the short‐chain fatty acid‐producing bacteria and the proliferation of TC. The gut microbiota alteration also affects the host metabolic pathways of TC patients (Figure 6).

**TABLE 2:** Recent studies on the relationship between gut microbiota and TC/TN.

TC complications	Samples used	Samples type	Methods used	Augmented bacterial phyla	Reduced bacterial phyla	Abundant genera	Reduced genera	Comments	References
Grave's disease (GD)	27 GD patients (10 males and 17 females), 11 healthy subjects (4 males and 7 females)	Stool	16S rRNA sequencing	Actinobacteria, Cyanobacteria	Firmicutes, Bacteroidetes	*Bacteroides*, *Escherichia*‐*Shigella*, *Parasutterella*	*Prevotella*_9, *Dialister*	Gut flora of GD patients was less diverse than healthy control	Ishaq et al. (2018)
Hashimoto's thyroiditis (HT)	28 HT patients and 16 matched healthy controls	Fecal	16S rRNA sequencing	Firmicutes	Bacteroidetes	*Fusicatenibacter*, *Blautia*, *Romboutsia*, *Dorea*, *Clostridium*_*sensu*_*stricto* 1	*Prevotella*_9, *Bacteroides*, *Fecalibacterium*, *Alloprevotella*, *Lachnoclostridium*, *Phascolarctobacterium*	HT patients have altered gut microbiota which is correlated with clinical parameters	Zhao et al. (2018)
Hashimoto's thyroiditis	45 Euthyroid, 18 Hypothyroid	Fecal	16S rRNA sequencing	ND	Bacteroidetes	*Phascolarctobacterium*	*Lachnospiraceae*_*incertae*_*sedis*, *Lactonifactor*, *Alistipes*	Thyroid peripheral homeostasis was sensitive to microbiota changes	Camilla Virili et al. (2021)
TC	90 TC samples	Stool	16S rRNA sequencing	Firmicutes, Bacteroidetes, Actinobacteria, Proteobacteria	ND	*Bacteroides, Lachnoclostridium*	*Prevotella*_9, *Collinsella*, *Faecalibacterium*, *Dorea*	Loss of the short‐chain fatty acid‐producing bacteria promoted TC	Yu et al. (2022)
Fine particulate matter (PM 2.5)	Stool sample from rat models	Stool	16S rRNA sequencing	Verrucomicrobiota, Elusimicrobiota, Patescibacteria, Desulfobacterota, Firmicutes	Cyanobacteria, Bacteroidetes, Proteobacteria	*Elusimicrobium*, *Muribaculum*, *Eubacterium*, *Parabacteroides*	*Prevotella*	PM2.5 exposure disturbed vital metabolic pathways related to thyroid toxicity	Dong et al. (2022)
Papillary TC	366 papillary TC and 42 healthy controls	Tissue	Whole‐transcriptome RNA‐sequencing	ND	ND	*Micrococcus luteus*, *Frankia*, *Anabaena* sp. K119, *Gammaproteobacteria bacterium*	*Trueperella*, *Stenotrophomonas*	Microbial dysbiosis lead to high levels of mutation and causing greater cancer severity	Gnanasekar et al. (2021)
TC and TN	74 (20 patients with TC, 18 with TN, and 36 healthy controls (HC)	Fecal	16S sequence	Firmicutes, Bacteroidetes	Proteobacteria	*Neisseria, Streptococcus*	*Butyricimona*s, *Lactobacillus*	Microbial richness was dominantly higher in both the TC and TN group	Zhang, Zhang, et al. (2019)
TN	196 patients with TN and 283 control	Stool	Whole‐genome shotgun sequencing	ND	ND	*Butyrivibrio unclassified*, *Bacteroides plebeius*, *Coprococcus comes*, *Coprococcus catus*, *Roseburia hominis*	*Bacteroides ovatus*, *Eggerthella unclassified*	Gut‐thyroid link is mediated via microbial nutrition metabolism	Li et al. (2021)
Euthyroid TC	16 TC patients and 10 from healthy subjects	Fecal	16S rRNA gene	Firmicutes, Verrucomicrobia	Bacteroidetes	*Escherichia‐Shigella, Akkermansia_coprostanoligenes, Dorea, Subdoligranulum Ruminococcus_2*	*Prevotella_9, Bacteroides, Klebsiella*	Euthyroid TC patients have significant gut microbial dysbiosis	Ishaq et al. (2022)

The whole‐genome sequencing of gut microbiome of 196 TN patients and 283 controls revealed that high‐grade TN patients have high amino acid degradation and less butyrate production. Thyrotropin‐releasing hormone decreased the abundance of butyrate‐producing microbes and l‐histidine metabolism pathways (Li et al., 2021). Microbial diversity differed significantly in papillary TC and advanced T1/T2 PTC female and male patients. The study indicated that tumor‐resident microbes play an eminent role in the progression of papillary TC (Yuan et al., 2022). *Clostridium subterminale*, a gut microbe was detected in the blood of a metastatic gastrointestinal adenocarcinoma patient, indicating the carcinogenic effect of Clostridiaceae have carcinogenic species (Trapani et al., 2018).

## 5 TC TREATMENT

### 5.1 TC surgery

Thyroid surgery is among the common procedures opted for TC. Open‐neck approach is suggested for malignant thyroid complications. In TC surgery, the thyroid can be retrieved through an anterior cervical slit, or by remote techniques (Pace‐Asciak et al., 2022). Open surgery method offers high revelation to the parathyroid glands, and vasculature, and facilitates access to the central or lateral neck (Haugen et al., 2016). While for well‐differentiated TC, completeness of surgical resection often leads to a remarkable prognosis (Shah et al., 2003). Intermediate TC includes aggressive histology, vascular invasion minor extrathyroidal extension, and >5 cm lymph nodes. High‐risk TC showed gross extrathyroidal extension, distant metastases, incomplete tumor resection, or lymph nodes >3 cm. Both intermediate and high‐risk TC can opt open approach as the cornerstone for surgical removal of the thyroid, central compartment, and or lateral neck compartments.

### 5.2 Lobectomy

The removal of one‐half of the thyroid gland is called a thyroid lobectomy. Lobectomy was recommended by the 2015 American Thyroid Association guidelines for surgical treatment of low‐risk differentiated TC and central neck dissections (Haugen et al., 2016). Low‐risk differentiated TC includes tumors between 1 and 4 cm and follicular or papillary TC without extrathyroidal extensions (Conroy et al., 2022). Patients treated with lobectomy showed lesser chances to be hospitalized or having hypoparathyroidism postoperatively. However, a thyroid lobectomy is not suggested for patients with nodules on both sides of the gland. Lobectomy for low‐risk differentiated TC may also increase the chances of complete thyroidectomy. In a retrospective study of 149 patients with differentiated TC (low‐risk), 20% of the patients who underwent lobectomy later were recommended for completion thyroidectomy (Kluijfhout et al., 2017).

### 5.3 Thyroidectomy

The total or complete thyroidectomy (with or without central neck dissection) is a widely suggested treatment for >4 cm tumors (Conroy et al., 2022). Conventional surgical thyroidectomy which includes an incision under the breast to bilateral axillo‐breast and axilla has been replaced with remote access techniques for hiding scars (Berber et al., 2016). Recently, transoral endoscopic thyroidectomy vestibular (TOETVA) has allowed a three‐dimensional magnified view through the endoscope and access to the thyroid with little soft tissue dissection (Russell & Razavi, 2021). TOETVA does not include cutaneous scars and offers precise control of the instruments. However, the high cost of instruments and their maintenance is still a limiting factor (Kandil et al., 2016). TOETVA was effective and safe for differentiated TC (low‐risk) patients with tumors of <2 cm without extracapsular spread (Chai et al., 2017).

### 5.4 Radioactive iodine treatment and radiation therapy

Radioactive iodine treatment (RIT) is applicable for the selected patients based on the reappearance and death rate of TC, as per the American Joint Committee on Cancer Union for International Cancer Control Tumor, Node, Metastasis (AJCC/TNM) staging system. RIT is also considered to be an adjuvant followed after surgery to increase survival (Valerio et al., 2022). RIT also includes relic excision and treatment of metastatic diseases. The effectiveness of RIT on differential TC treatment depends on the level of serum thyrotropin which can either be raised endogenously or by exogenous administration of rh serum thyrotropin (Leenhardt et al., 2019). However, there is no evidence on stimulation approaches for accessary RIT in intermediate risk differential TC patients. The oral administration of ^131^I significantly reduced Firmicutes to Bacteroides (F/B) ratio and altered gut microbiome‐related metabolic pathways leading to gut‐microbiota dysbiosis (Zheng et al., 2023).

### 5.5 External radiation therapy

External radiation therapy is applicable for the treatment of both differentiated and medullary TC (Brierley & Sherman, 2012). A combination of surgery with radiation therapy is more effective than radiations alone in anaplastic TC (Haigh et al., 2001). The effectiveness of radiation therapy relies on the adequate dosage to the region at risk while minimized exposure to surrounding critical structures (Terezakis & Lee, 2010). Adjuvant external beam radiation therapy was effective for local control in papillary TC invading the trachea with tolerable complications (Kim, 2015). The combination of radiation therapy with Lenvatinib significantly reduced the TC growth by inducing apoptosis. Radiations (3 Gy) also increased the uptake of Lenvatinib inside the cancer cells (Suzuki et al., 2021). The external radiations influence the gut microbes bidirectionally as radiotherapy can disrupt the microbiome and influence the effectiveness of the anticancer treatments (Liu et al., 2021). The long‐term changes in gut microbiota evaluation post external therapy demonstrated an increase in the abundance of *Bacteroidia* and a decrease in *Clostridia* diversity in 5 C57BL/6 mice, compared to control (Zhao et al., 2019).

### 5.6 Hormone therapy

Thyroid hormone therapy is a recommended treatment for patients who have undergone thyroidectomy or lobectomy to balance the normal TSH level. TSH can influence the proliferation of TC, thus, in some patients, suppression of TSH is recommended (Grani et al., 2019). The levothyroxine‐mediated TSH suppression keeps serum TSH levels within the standard limit (Lamartina et al., 2017). The TSH suppression therapy also showed insignificant results in some intermediate‐ and high‐risk papillary TC patients (Tian et al., 2019). Long‐term TSH suppression therapy can also lead to cardiovascular disease risk (such as myocardial strain and impaired diastolic function), osteoporosis, and chronic thyrotoxicosis (Biondi et al., 1996; Do Cao & Wémeau, 2015; Kim, Jeon, et al., 2019).

### 5.7 Chemotherapy and treatment with targeted drugs for TC

The targeted drug treatment includes specific gene inhibitors to control the TC. For instance, multikinase inhibitors (lenvatinib and sorafenib) were used against advanced radioactive iodine‐refractory differentiated TC. For BRAF gene mutation (including BRAFV600E mutated ATC), combined MEK and BRAF inhibitors (dabrafenib and trametinib) were approved to improve the clinical symptoms in TC patients (Subbiah et al., 2020). Second‐generation tyrosine kinase inhibitors (TKIs) have lately been established to target an explicit oncogene: RET gene (selpercatinib, pralsetinib) and NTRK gene (larotrectinib, entrectinib) fusions for metastatic TC (Owen et al., 2019). The efficacy of chemotherapy also relies on gut microbes which modulate the host response to chemotherapeutic drugs. Gut microbial composition regulates the facilitation of drug efficacy, abrogation of anticancer effects, and mediation of toxicity (Alexander et al., 2017).

### 5.8 Targeted treatment

The progress in understanding of gene and genetic changes in TC resulted in the discovery of various targeted therapies with high clinical efficiency. These targeted modifications include fusion of NTRK (neurotrophic tyrosine receptor kinase) gene with the tropomyosin receptor kinase inhibitors entrectinib and larotrectinib (Capdevila et al., 2022). Administration of Vemurafenib is also permitted by the European Medicines Agency for the treatment of BRAF V600E mutations in patients showing unresectable or metastatic melanoma. Selpercatinib is another specific RET kinase inhibitor approved by the EMA for adult patients with advanced RET fusion‐positive TC (Santoro et al., 2020).

### 5.9 Immunotherapy for TC

Immunotherapy for TC involves the use of immune checkpoint inhibitors (ICIs) that improve the immune system by inhibiting the binding of cancer checkpoint receptors to their ligands. The most commonly used ICI are Cytotoxic T‐lymphocyte antigen‐4 antagonist, PD‐1 ligand (PD‐L1) antagonist, programmed cell death protein‐1, and (PD‐1) antagonist (Antonelli et al., 2018). In some cases, resistance to KIs has been observed due to the outflow phenomenon due to the cross‐talk between tumorigenesis pathways and the cell surface upregulation of tyrosine kinase receptors (Naoum et al., 2018). A retrospective study investigating the effect of KIs at the time of anaplastic TC progression suggested the addition of pembrolizumab as a recovering therapy (Iyer et al., 2018).

## 6 PROBIOTICS EFFECT ON TC

**FIGURE 7:**
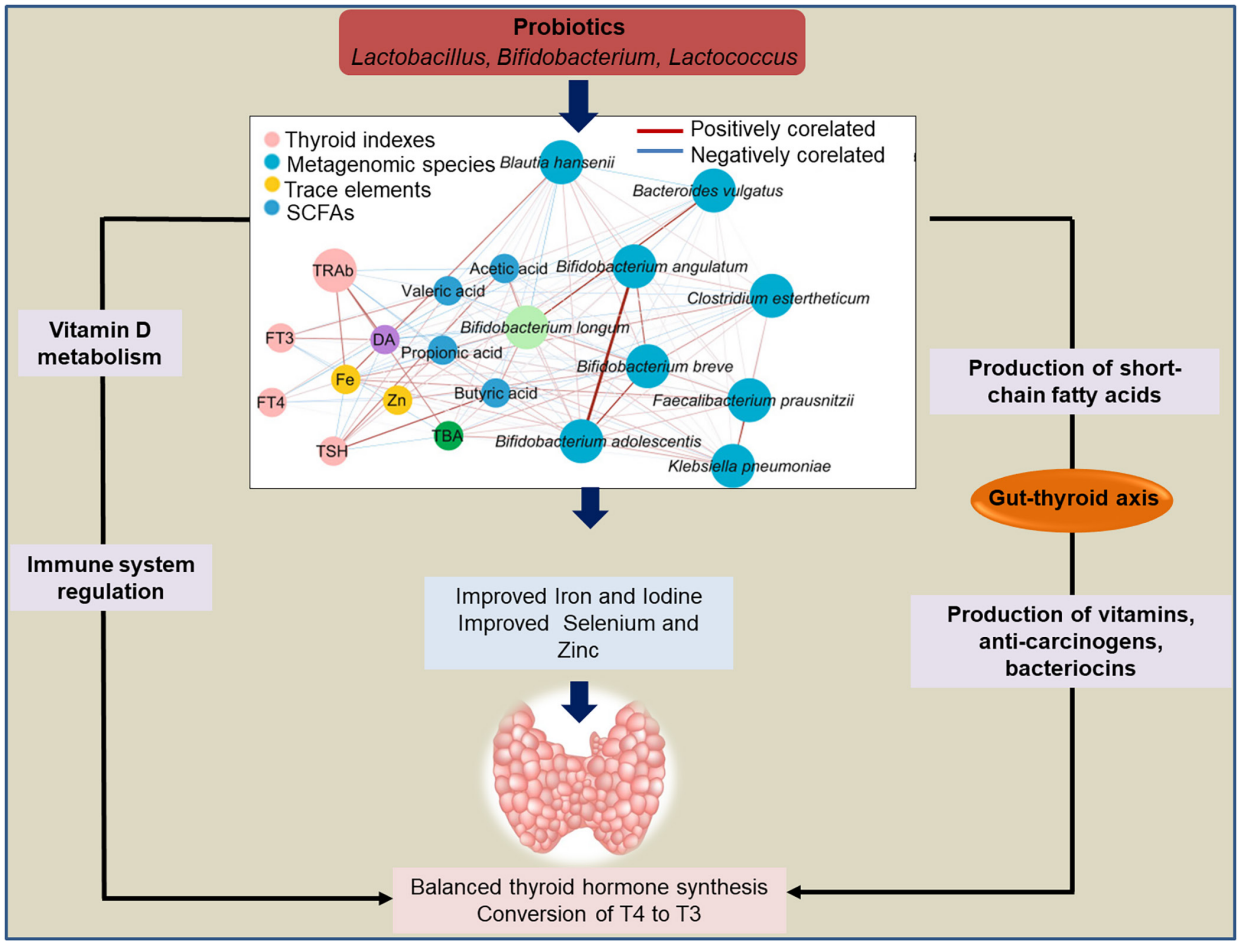
Probiotics affect the enhancement of beneficial gut‐microbiota and thyroid function through the gut‐thyroid axis (Knezevic et al., 2020).

The application of probiotics could favor the enrichment of beneficial gut microbes that can indirectly influence TC (Fröhlich & Wahl, 2019). In TC, the *Lactobacillus* and *Bifidobacteria* are often reduced. Supplementation of *L. reuteri* in mice model increased T4 count by triggering interleukine‐10 and subsequently enhanced T‐regulatory cells (Virili et al., 2018). Supplementation of *Lactobacillus‐*based probiotics to broiler chickens improved the count of T3 and T4 (Sohail et al., 2010). Probiotics lower the serum hormonal fluctuations and also regulate the iodothyronines deconjugation through bacterial enzymes (such as sulfatases and β‐glucuronidases) (Knezevic et al., 2020). Probiotics can accumulate trace elements (such as zine, selenium, and copper) and integrate them into essential organic compounds. These trace elements are eminent for thyroid function (Figure 7). Probiotics might decrease the frequency of complications in TC patients by modifying the oral and gut microbiota. Probiotics supplementation composed of *B. infantis*, *L. acidophilus*, *E. faecalis*, and *B. cereus* reduced the TC complications and restored the oral and gut microbiota (Lin et al., 2022). Probiotics decreased the oral *Prevotella*_9, *Fusobacterium*, *Haemophilus*, and *Lautropia*, while increased the gut *Holdemanella*, *Coprococcus*_2, and *Enterococcu*s. Probiotics also reduced the abundance of oral microbiota which is positively correlated with mouth cancer (Lin et al., 2022).

The consumption of probiotics regulates the gut microbiota and the metabolites which interact with the neurotransmitters through the gut‐thyroid axis to improve thyroid function (Huo et al., 2021). Another approach to improve the gut microbiota is the incorporation of prebiotics in the diet which can further improve thyroid functioning and prevent chances of TC (Yasmin et al., 2015). Prebiotics induces specific changes in the gastrointestinal microbiota by altering the microbial ecology and fermentation profiles (Rehman et al., 2008). Supplementation of mannooligosaccharide prebiotic enhanced the growth of *Lactobacillus* and *Bifidobacterium*, which play essential roles in thyroid functioning (Baurhoo et al., 2007). Prebiotics can also stimulate the immune system which has an indirect effect on thyroid hormones and the restoration of beneficial microbes. A combination of pre‐and probiotics can have a high influence on improving gut microbial consortium and thyroid gland.

## 7 CONCLUSION AND FUTURE PROSPECTIVE

TC is among the common endocrine malignancy which is directly affected by lifestyle changes (such as nourishment, alcohol intake, and BMI), while indirectly by the oral and gut microbiota. The iodine and Se deficiency stimulated the development of TC. Reduced TC count and variations in TSH level due to alcohol intake and high BMI, respectively, also reported to promote TC. TC can increase the count of *Alloprevotella*, *Anaeroglobus*, and *Acinetobacter* genera in the oral cavity. While, the most abundantly reported gut microbial genera during TC were *Bacteroides*, *Neisseria*, *Streptococcus*, and *Clostridium*. The gut microbiota also influences the efficacy of TC treatments (such as radiotherapy and chemotherapy). Supplementation of probiotics can improve the beneficial gut microbes such as *Lactobacilli* and *Bifidobacterium*. However, the underlying mechanism of probiotics interaction with TG function has not been clearly illustrated and requires more in‐depth metabolomics‐based research.

## AUTHOR CONTRIBUTIONS

**Yao Kun:** Conceptualization (equal); data curation (equal); formal analysis (equal); investigation (equal); validation (equal); visualization (equal). **Wei Xiaodong:** Conceptualization (equal); data curation (equal); acquisition (equal); methodology (equal); supervision (equal); validation (equal); visualization (equal). **Wang Haijun:** Data curation (equal); formal analysis (equal); investigation (equal); methodology (equal); validation (equal); visualization (equal). **Nie Xiazi:** Data curation (equal); formal analysis (equal); investigation (equal); validation (equal); visualization (equal). **Qiang Dai:** Formal analysis (equal); investigation (equal); validation (equal); visualization (equal).

## FUNDING INFORMATION

The work was supported by the Research Fund of Gansu Provincial Hospital (20GSSY1‐16 and 20GSSY4‐18).

## CONFLICT OF INTEREST STATEMENT

The authors declare no competing interests.

## ETHICS STATEMENT

Not applicable.

## CONSENT FOR PUBLICATION

All the authors agree to publish this article.

## Data Availability

Data will be made available on request.

## References

[fsn33538-bib-0001] Accardo, G. , Conzo, G. , Esposito, D. , Gambardella, C. , Mazzella, M. , Castaldo, F. , & Pasquali, D. (2017). Genetics of medullary thyroid cancer: An overview. International Journal of Surgery, 41(Suppl 1), S2–S6. 10.1016/j.ijsu.2017.02.064 28506408

[fsn33538-bib-0002] Afzaal, M. , Saeed, F. , Shah, Y. A. , Hussain, M. , Rabail, R. , Socol, C. T. , Hassoun, A. , Pateiro, M. , Lorenzo, J. M. , Rusu, A. V. , & Aadil, R. M. (2022). Human gut microbiota in health and disease: Unveiling the relationship. Frontiers in Microbiology, 13, 999001. 10.3389/fmicb.2022.999001 36225386 PMC9549250

[fsn33538-bib-0003] Alexander, J. L. , Wilson, I. D. , Teare, J. , Marchesi, J. R. , Nicholson, J. K. , & Kinross, J. M. (2017). Gut microbiota modulation of chemotherapy efficacy and toxicity. Nature Reviews Gastroenterology & Hepatology, 14(6), 356–365. 10.1038/nrgastro.2017.20 28270698

[fsn33538-bib-0004] Alhashemi, A. , Jones, J. M. , Goldstein, D. P. , Ezzat, S. , & Sawka, A. M. (2019). Re: Quality of life and symptom impact of thyroid cancer: A cross‐sectional survey of Canadian patients. Surgery, 166(5), 948–949. 10.1016/j.surg.2018.09.018 30385121

[fsn33538-bib-0005] Ali, N. H. , & Majeed, A. A. (2022). Thyroid hormone concentration and receptor. Egyptian Academic Journal of Biological Sciences, B. Zoology, 14(1), 221–230.

[fsn33538-bib-0006] Antonelli, A. , Ferrari, S. M. , & Fallahi, P. (2018). Current and future immunotherapies for thyroid cancer. Expert Review of Anticancer Therapy, 18(2), 149–159. 10.1080/14737140.2018.1417845 29241377

[fsn33538-bib-0007] Aoun, E. G. , Lee, M. R. , Haass‐Koffler, C. L. , Swift, R. M. , Addolorato, G. , Kenna, G. A. , & Leggio, L. (2015). Relationship between the thyroid axis and alcohol craving. Alcohol and Alcoholism, 50(1), 24–29. 10.1093/alcalc/agu085 25433251 PMC4266183

[fsn33538-bib-0008] Aydin, L. Y. , Aydin, Y. , Besir, H. F. , Demirin, H. , Yildirim, H. , Onder, E. , Dumlu, T. , & Celbek, G. (2011). Effect of smoking intensity on thyroid volume, thyroid nodularity and thyroid function: The Melen study. Minerva Endocrinologica, 36(4), 273–280. 22322651

[fsn33538-bib-0009] Balhara, Y. P. , & Deb, K. S. (2013). Impact of alcohol use on thyroid function. Indian Journal of Endocrinology and Metabolism, 17(4), 580–587. 10.4103/2230-8210.113724 23961472 PMC3743356

[fsn33538-bib-0010] Barrea, L. , Gallo, M. , Ruggeri, R. M. , Di Giacinto, P. , Sesti, F. , Prinzi, N. , Adinolfi, V. , Barucca, V. , Renzelli, V. , Muscogiuri, G. , Colao, A. , Baldelli, R. , & E.O.L.O. Group . (2021). Nutritional status and follicular‐derived thyroid cancer: An update. Critical Reviews in Food Science and Nutrition, 61(1), 25–59. 10.1080/10408398.2020.1714542 31997660

[fsn33538-bib-0011] Baurhoo, B. , Phillip, L. , & Ruiz‐Feria, C. A. (2007). Effects of purified lignin and mannan oligosaccharides on intestinal integrity and microbial populations in the ceca and litter of broiler chickens. Poultry Science, 86(6), 1070–1078. 10.1093/ps/86.6.1070 17495075

[fsn33538-bib-0012] Bednarczuk, N. , Milner, A. , & Greenough, A. (2020). The role of maternal smoking in sudden fetal and infant death pathogenesis. Frontiers in Neurology, 11, 586068. 10.3389/fneur.2020.586068 33193050 PMC7644853

[fsn33538-bib-0013] Berber, E. , Bernet, V. , Fahey, T. J., 3rd , Kebebew, E. , Shaha, A. , Stack, B. C., Jr. , Stang, M. , Steward, D. L. , Terris, D. J. , & American Thyroid Association Surgical Affairs Committee . (2016). American Thyroid Association Statement on remote‐access thyroid surgery. Thyroid, 26(3), 331–337. 10.1089/thy.2015.0407 26858014 PMC4994052

[fsn33538-bib-0015] Biondi, B. , Fazio, S. , Cuocolo, A. , Sabatini, D. , Nicolai, E. , Lombardi, G. , Salvatore, M. , & Saccà, L. (1996). Impaired cardiac reserve and exercise capacity in patients receiving long‐term thyrotropin suppressive therapy with levothyroxine. The Journal of Clinical Endocrinology and Metabolism, 81(12), 4224–4228. 10.1210/jcem.81.12.8954019 8954019

[fsn33538-bib-0016] Brierley, J. , & Sherman, E. (2012). The role of external beam radiation and targeted therapy in thyroid cancer. Seminars in Radiation Oncology, 22(3), 254–262. 10.1016/j.semradonc.2012.03.010 22687950

[fsn33538-bib-0017] Canaris, G. J. , Manowitz, N. R. , Mayor, G. , & Ridgway, E. C. (2000). The Colorado thyroid disease prevalence study. Archives of Internal Medicine, 160(4), 526–534. 10.1001/archinte.160.4.526 10695693

[fsn33538-bib-0018] Capdevila, J. , Awada, A. , Führer‐Sakel, D. , Leboulleux, S. , & Pauwels, P. (2022). Molecular diagnosis and targeted treatment of advanced follicular cell‐derived thyroid cancer in the precision medicine era. Cancer Treatment Reviews, 106, 102380. 10.1016/j.ctrv.2022.102380 35305441

[fsn33538-bib-0019] Chai, Y. J. , Chung, J. K. , Anuwong, A. , Dionigi, G. , Kim, H. Y. , Hwang, K. T. , Heo, S. C. , Yi, K. H. , & Lee, K. E. (2017). Transoral endoscopic thyroidectomy for papillary thyroid microcarcinoma: Initial experience of a single surgeon. Annals of Surgical Treatment and Research, 93(2), 70–75. 10.4174/astr.2017.93.2.70 28835882 PMC5566749

[fsn33538-bib-0020] Cherella, C. E. , & Wassner, A. J. (2023). Pediatric thyroid cancer: Recent developments. Best Practice & Research Clinical Endocrinology & Metabolism, 37(1), 101715. 10.1016/j.beem.2022.101715 36404191

[fsn33538-bib-0021] Conroy, P. C. , Wilhelm, A. , Calthorpe, L. , Ullmann, T. M. , Davis, S. , Huang, C.‐Y. , Shen, W. T. , Gosnell, J. , Duh, Q.‐Y. , Roman, S. , & Sosa, J. A. (2022). Endocrine surgeons are performing more thyroid lobectomies for low‐risk differentiated thyroid cancer since the 2015 ATA guidelines. Surgery, 172(5), 1392–1400. 10.1016/j.surg.2022.06.031 36002375

[fsn33538-bib-0022] Dai, H. , Zhang, L. , Han, X. , Zhao, H. , Guo, J. , Li, Z. , & Yang, A. (2020). Body mass index (BMI) is associated with serum thyroid‐stimulating hormone (TSH) level in infertile women: A cross‐sectional study. Endocrine Journal, 67(9), 923–928. 10.1507/endocrj.EJ19-0441 32418923

[fsn33538-bib-0023] de Lima Junior, N. C. , Camilo, J. F. , do Carmo, P. R. , de Andrade, M. N. , Braz, B. F. , Santelli, R. E. , de Brito Gitirana, L. , Ferreira, A. C. F. , de Carvalho, D. P. , Miranda‐Alves, L. , & Dias, G. R. M. (2021). Subacute exposure to lead promotes disruption in the thyroid gland function in male and female rats. Environmental Pollution, 274, 115889. 10.1016/j.envpol.2020.115889 33223335

[fsn33538-bib-0024] de Souza Dos Santos, M. C. , Goncalves, C. F. , Vaisman, M. , Ferreira, A. C. , & de Carvalho, D. P. (2011). Impact of flavonoids on thyroid function. Food and Chemical Toxicology, 49(10), 2495–2502. 10.1016/j.fct.2011.06.074 21745527

[fsn33538-bib-0025] Divi, R. L. , Chang, H. C. , & Doerge, D. R. (1997). Anti‐thyroid isoflavones from soybean: Isolation, characterization, and mechanisms of action. Biochemical Pharmacology, 54(10), 1087–1096. 10.1016/s0006-2952(97)00301-8 9464451

[fsn33538-bib-0026] Do Cao, C. , & Wémeau, J. L. (2015). Risk‐benefit ratio for TSH‐ suppressive levothyroxine therapy in differentiated thyroid cancer. Annales d'endocrinologie, 76(1 Suppl 1), 1S47–1S52. 10.1016/s0003-4266(16)30014-2 26826483

[fsn33538-bib-0027] Dong, T. , Zhao, F. , Yuan, K. , Zhu, X. , Wang, N. , Xia, F. , Lu, Y. , & Huang, Z. (2021). Association between serum thyroid‐stimulating hormone levels and salivary microbiome shifts. Frontiers in Cellular and Infection Microbiology, 11, 603291. 10.3389/fcimb.2021.603291 33718264 PMC7952758

[fsn33538-bib-0028] Dong, X. , Yao, S. , Deng, L. , Li, H. , Zhang, F. , Xu, J. , Li, Z. , Zhang, L. , Jiang, J. , & Wu, W. (2022). Alterations in the gut microbiota and its metabolic profile of PM2.5 exposure‐induced thyroid dysfunction rats. Science of the Total Environment, 838, 156402. 10.1016/j.scitotenv.2022.156402 35660575

[fsn33538-bib-0029] Du, L. , Li, R. , Ge, M. , Wang, Y. , Li, H. , Chen, W. , & He, J. (2019). Incidence and mortality of thyroid cancer in China, 2008‐2012. Chinese Journal of Cancer Research, 31(1), 144–151. 10.21147/j.issn.1000-9604.2019.01.09 30996572 PMC6433579

[fsn33538-bib-0120] Duntas, L. H. , & Stathatos, N. (2015). Toxic chemicals and thyroid function: hard facts and lateral thinking. Reviews in Endocrine & Metabolic Disorders, 16, 311–318. 10.1007/s11154-016-9331-x 26801661

[fsn33538-bib-0030] El‐Fadeli, S. , Bouhouch, S. , Skalny, A. V. , Barkouch, Y. , Pineau, A. , Cherkaoui, M. , & Sedki, A. (2016). Effects of imbalance in trace element on thyroid gland from Moroccan children. Biological Trace Element Research, 170(2), 288–293. 10.1007/s12011-015-0485-2 26315305

[fsn33538-bib-0031] Fenneman, A. C. , & Bruinstroop, E. (2023). A comprehensive review of thyroid hormone metabolism in the gut and its clinical implications. Thyroid, 33(1), 32–44. 10.1089/thy.2022.0491 36322786

[fsn33538-bib-0032] Fernández‐García, V. , González‐Ramos, S. , Martín‐Sanz, P. , Laparra, J. M. , & Boscá, L. (2021). Beyond classic concepts in thyroid homeostasis: Immune system and microbiota. Molecular and Cellular Endocrinology, 533, 111333. 10.1016/j.mce.2021.111333 34048865

[fsn33538-bib-0033] Filis, P. , Hombach‐Klonisch, S. , Ayotte, P. , Nagrath, N. , Soffientini, U. , Klonisch, T. , O'Shaughnessy, P. , & Fowler, P. A. (2018). Maternal smoking and high BMI disrupt thyroid gland development. BMC Medicine, 16(1), 194. 10.1186/s12916-018-1183-7 30348172 PMC6198368

[fsn33538-bib-0034] Fröhlich, E. , & Wahl, R. (2019). Microbiota and thyroid interaction in health and disease. Trends in Endocrinology & Metabolism, 30(8), 479–490. 10.1016/j.tem.2019.05.008 31257166

[fsn33538-bib-0035] Gharib, H. (2018). Does iodine cause thyroid cancer? Acta Endocrinologica, 14(4), 525–526. 10.4183/aeb.2018.525 31149307 PMC6516421

[fsn33538-bib-0126] Gnanasekar, A. , Castaneda, G. , Iyangar, A. , Magesh, S. , Perez, D. , Chakladar, J. , Li, T. , Bouvet, M. , Chang, E. Y. , & Ongkeko, W. M. (2021). The intratumor microbiome predicts prognosis across gender and subtypes in papillary thyroid carcinoma. Computational and Structural Biotechnology Journal, 19, 1986–1997. 10.1016/j.csbj.2021.03.032 33995898 PMC8085784

[fsn33538-bib-0036] Gordon, H. V. (2012). Complexities in the diagnosis and treatment of thyroid cancer: Discussions, observations, research and public policy . Medicine.

[fsn33538-bib-0037] Grani, G. , Ramundo, V. , Verrienti, A. , Sponziello, M. , & Durante, C. (2019). Thyroid hormone therapy in differentiated thyroid cancer. Endocrine, 66(1), 43–50. 10.1007/s12020-019-02051-3 31617165

[fsn33538-bib-0038] Gruppen, E. G. , Kootstra‐Ros, J. , Kobold, A. M. , Connelly, M. A. , Touw, D. , Bos, J. H. J. , Hak, E. , Links, T. P. , Bakker, S. J. L. , & Dullaart, R. P. F. (2020). Cigarette smoking is associated with higher thyroid hormone and lower TSH levels: The PREVEND study. Endocrine, 67(3), 613–622. 10.1007/s12020-019-02125-2 31707605 PMC7054375

[fsn33538-bib-0039] Gumulec, J. , Masarik, M. , Adam, V. , Eckschlager, T. , Provaznik, I. , & Kizek, R. (2014). Serum and tissue zinc in epithelial malignancies: A meta‐analysis. PLoS One, 9(6), e99790. 10.1371/journal.pone.0099790 24941118 PMC4062461

[fsn33538-bib-0040] Habib, A. , Molayemat, M. , & Habib, A. (2020). Elevated serum TSH concentrations are associated with higher BMI Z‐scores in southern Iranian children and adolescents. Thyroid Research, 13, 9. 10.1186/s13044-020-00084-9 32547643 PMC7293126

[fsn33538-bib-0041] Haigh, P. I. , Ituarte, P. H. , Wu, H. S. , Treseler, P. A. , Posner, M. D. , Quivey, J. M. , Duh, Q. Y. , & Clark, O. H. (2001). Completely resected anaplastic thyroid carcinoma combined with adjuvant chemotherapy and irradiation is associated with prolonged survival. Cancer, 91(12), 2335–2342. 11413523

[fsn33538-bib-0042] Haugen, B. R. , Alexander, E. K. , Bible, K. C. , Doherty, G. M. , Mandel, S. J. , Nikiforov, Y. E. , Pacini, F. , Randolph, G. W. , Sawka, A. M. , Schlumberger, M. , Schuff, K. G. , Sherman, S. I. , Sosa, J. A. , Steward, D. L. , Tuttle, R. M. , & Wartofsky, L. (2016). 2015 American Thyroid Association management guidelines for adult patients with thyroid nodules and differentiated thyroid cancer: The American Thyroid Association Guidelines Task Force on Thyroid Nodules and Differentiated Thyroid Cancer. Thyroid, 26(1), 1–133. 10.1089/thy.2015.0020 26462967 PMC4739132

[fsn33538-bib-0043] Hermann, D. , Heinz, A. , & Mann, K. (2002). Dysregulation of the hypothalamic‐pituitary‐thyroid axis in alcoholism. Addiction, 97(11), 1369–1381. 10.1046/j.1360-0443.2002.00200.x 12410778

[fsn33538-bib-0044] Hou, J. , Tang, Y. , Chen, Y. , & Chen, D. (2021). The role of the microbiota in Graves' disease and Graves' orbitopathy. Frontiers in Cellular and Infection Microbiology, 11, 739707. 10.3389/fcimb.2021.739707 35004341 PMC8727912

[fsn33538-bib-0045] Hou, T. , Wang, Q. , Dai, H. , Hou, Y. , Zheng, J. , Wang, T. , & Bi, Y. (2023). Interactive association between gut microbiota and thyroid cancer: A Mendelian randomization and systematic review. *medRxiv* . 10.1101/2023.03.27.23287832 38051644

[fsn33538-bib-0046] Huo, D. , Cen, C. , Chang, H. , Ou, Q. , Jiang, S. , Pan, Y. , Chen, K. , & Zhang, J. (2021). Probiotic *Bifidobacterium longum* supplied with methimazole improved the thyroid function of Graves' disease patients through the gut‐thyroid axis. Communications Biology, 4(1), 1046. 10.1038/s42003-021-02587-z 34493790 PMC8423791

[fsn33538-bib-0047] Iglesias, M. L. , Schmidt, A. , Ghuzlan, A. A. , Lacroix, L. , Vathaire, F. , Chevillard, S. , & Schlumberger, M. (2017). Radiation exposure and thyroid cancer: A review. Archives of Endocrinology and Metabolism, 61(2), 180–187. 10.1590/2359-3997000000257 28225863 PMC10118869

[fsn33538-bib-0127] Ishaq, H. M. , Mohammad, I. S. , Hussain, R. , Parveen, R. , Shirazi, J. H. , Fan, Y. , Shahzad, M. , Hayat, K. , Li, H. , Ihsan, A. , Muhammad, K. S. , Usman, M. , Zhang, S. , Yuan, L. , Ullah, S. , Paiva‐Santos, A. C. , & Xu, J. (2022). Gut‐Thyroid axis: How gut microbial dysbiosis associated with euthyroid thyroid cancer. Journal of Cancer, 13, 2014–2028. 10.7150/jca.66816 35399732 PMC8990431

[fsn33538-bib-0123] Ishaq, H. M. , Shahzad, M. , Wu, X. , Ma, C. , & Xu, J. (2018). Gut Microbe Analysis between Asthma Patients and Healthy Volunteers in Shaanxi Province, Xi'an, China. Pakistan Journal of Zoology, 50, 165–173. 10.17582/journal.pjz/2018.50.1.165.173

[fsn33538-bib-0048] Iyer, P. C. , Dadu, R. , Gule‐Monroe, M. , Busaidy, N. L. , Ferrarotto, R. , Habra, M. A. , Zafereo, M. , Williams, M. D. , Gunn, G. B. , Grosu, H. , Skinner, H. D. , Sturgis, E. M. , Gross, N. , & Cabanillas, M. E. (2018). Salvage pembrolizumab added to kinase inhibitor therapy for the treatment of anaplastic thyroid carcinoma. Journal for Immunotherapy of Cancer, 6(1), 68. 10.1186/s40425-018-0378-y 29996921 PMC6042271

[fsn33538-bib-0049] Jain, R. B. (2014). Thyroid function and serum copper, selenium, and zinc in general US population. Biological Trace Element Research, 159(1–3), 87–98. 10.1007/s12011-014-9992-9 24789479

[fsn33538-bib-0050] Jiao, J. , Zheng, Y. , Zhang, Q. , Xia, D. , Zhang, L. , & Ma, N. (2022). Saliva microbiome changes in thyroid cancer and thyroid nodules patients. Frontiers in Cellular and Infection Microbiology, 12. 10.3389/fcimb.2022.989188 PMC9403763 36034695

[fsn33538-bib-0051] Kandil, E. , Hammad, A. Y. , Walvekar, R. R. , Hu, T. , Masoodi, H. , Mohamed, S. E. , Deniwar, A. , & Stack, B. C., Jr. (2016). Robotic thyroidectomy versus nonrobotic approaches: A meta‐analysis examining surgical outcomes. Surgical Innovation, 23(3), 317–325. 10.1177/1553350615613451 26525401

[fsn33538-bib-0052] Kim, E. H. , Jeon, Y. K. , Pak, K. , Kim, I. J. , Kim, S. J. , Shin, S. , Kim, B. H. , Kim, S. S. , Lee, B.‐J. , Lee, J.‐G. , Goh, T. S. , & Kim, K. (2019). Effects of thyrotropin suppression on bone health in menopausal women with total thyroidectomy. Journal of Bone Metabolism, 26(1), 31–38. 10.11005/jbm.2019.26.1.31 30899722 PMC6416151

[fsn33538-bib-0053] Kim, S.‐J. , Kim, M. J. , Yoon, S. G. , Myong, J. P. , Yu, H. W. , Chai, Y. J. , Choi, J. Y. , & Lee, K. E. (2019). Impact of smoking on thyroid gland: Dose‐related effect of urinary cotinine levels on thyroid function and thyroid autoimmunity. Scientific Reports, 9, 4213. 10.1038/s41598-019-40708-1 30862792 PMC6414657

[fsn33538-bib-0054] Kim, Y. S. (2015). The role of adjuvant external beam radiation therapy for papillary thyroid cancer invading the trachea: A single‐institution study. International Journal of Radiation Oncology, Biology, Physics, 93(3, Supplement), E351. 10.1016/j.ijrobp.2015.07.1441

[fsn33538-bib-0055] Kluijfhout, W. P. , Pasternak, J. D. , Drake, F. T. , Beninato, T. , Shen, W. T. , Gosnell, J. E. , Suh, I. , C, L. , & Duh, Q.‐Y. (2017). Complexities in the diagnosis and treatment of thyroid cancer: Discussions, observations, research and public policy. Surgery, 161(1), 127–133. 10.1016/j.surg.2016.05.056 27855968

[fsn33538-bib-0056] Knezevic, J. , Starchl, C. , Tmava Berisha, A. , & Amrein, K. (2020). Thyroid‐gut‐axis: How does the microbiota influence thyroid function? Nutrients, 12(6), 1769. 10.3390/nu12061769 32545596 PMC7353203

[fsn33538-bib-0057] Koukkou, E. G. , Roupas, N. D. , & Markou, K. B. (2017). Effect of excess iodine intake on thyroid on human health. Minerva Medica, 108(2), 136–146. 10.23736/s0026-4806.17.04923-0 28079354

[fsn33538-bib-0058] Kunc, M. , Gabrych, A. , & Witkowski, J. M. (2016). Microbiome impact on metabolism and function of sex, thyroid, growth and parathyroid hormones. Acta Biochimica Polonica, 63(2), 189–201. 10.18388/abp.2015_1093 26505128

[fsn33538-bib-0059] Lamartina, L. , Durante, C. , Lucisano, G. , Grani, G. , Bellantone, R. , Lombardi, C. P. , Pontecorvi, A. , Arvat, E. , Felicetti, F. , Zatelli, M. C. , Rossi, R. , Puxeddu, E. , Morelli, S. , Torlontano, M. , Crocetti, U. , Montesano, T. , Giubbini, R. , Orlandi, F. , Aimaretti, G. , … Filetti, S. (2017). Are evidence‐based guidelines reflected in clinical practice? An analysis of prospectively collected data of the Italian Thyroid Cancer Observatory. Thyroid, 27(12), 1490–1497. 10.1089/thy.2017.0299 29020892

[fsn33538-bib-0060] LeClair, K. , Bell, K. J. L. , Furuya‐Kanamori, L. , Doi, S. A. , Francis, D. O. , & Davies, L. (2021). Evaluation of gender inequity in thyroid cancer diagnosis: Differences by sex in US thyroid cancer incidence compared with a meta‐analysis of subclinical thyroid cancer rates at autopsy. JAMA Internal Medicine, 181(10), 1351–1358. 10.1001/jamainternmed.2021.4804 34459841 PMC8406211

[fsn33538-bib-0061] Leenhardt, L. , Leboulleux, S. , Bournaud, C. , Zerdoud, S. , Schvartz, C. , Ciappuccini, R. , Kelly, A. , Morel, O. , Dygai‐Cochet, I. , Rusu, D. , Chougnet, C. N. , Lion, G. , Eberlé‐Pouzeratte, M.‐C. , Catargi, B. , Kabir‐Ahmadi, M. , Feuillet, E. L. P. , & Taïeb, D. (2019). Recombinant thyrotropin vs levothyroxine withdrawal in 131I therapy of N1 thyroid cancer: A large matched cohort study (ThyrNod). The Journal of Clinical Endocrinology and Metabolism, 104(4), 1020–1028. 10.1210/jc.2018-01589 30398518

[fsn33538-bib-0062] Li, A. , Li, T. , Gao, X. , Yan, H. , Chen, J. , Huang, M. , Wang, L. , Yin, D. , Li, H. , Ma, R. , Zeng, Q. , & Ding, S. (2021). Gut microbiome alterations in patients with thyroid nodules. Frontiers in Cellular and Infection Microbiology, 11, 643968. 10.3389/fcimb.2021.643968 33791245 PMC8005713

[fsn33538-bib-0063] Lin, B. , Zhao, F. , Liu, Y. , Wu, X. , Feng, J. , Jin, X. , Yan, W. , Guo, X. , Shi, S. , Li, Z. , Liu, L. , Chen, H. , Wang, H. , Wang, S. , Lu, Y. , & Wei, Y. (2022). Randomized clinical trial: Probiotics alleviated oral‐gut microbiota dysbiosis and thyroid hormone withdrawal‐related complications in thyroid cancer patients before radioiodine therapy following thyroidectomy. Frontiers in Endocrinology, 13, 834674. 10.3389/fendo.2022.834674 35350100 PMC8958007

[fsn33538-bib-0064] Liu, J. , Liu, C. , & Yue, J. (2021). Radiotherapy and the gut microbiome: Facts and fiction. Radiation Oncology, 16(1), 9. 10.1186/s13014-020-01735-9 33436010 PMC7805150

[fsn33538-bib-0065] Lu, G. , Yu, X. , Jiang, W. , Luo, Q. , Tong, J. , Fan, S. , Chai, L. , Gao, D. , Qiao, T. , Wang, R. , Deng, C. , Lv, Z. , & Li, D. (2022). Alterations of gut microbiome and metabolite profiles associated with anabatic lipid dysmetabolism in thyroid cancer. Frontiers in Endocrinology, 13, 893164. 10.3389/fendo.2022.893164 35721748 PMC9204252

[fsn33538-bib-0066] Maki, K. A. , Kazmi, N. , Barb, J. J. , & Ames, N. (2021). The oral and gut bacterial microbiomes: Similarities, differences, and connections. Biological Research for Nursing, 23(1), 7–20. 10.1177/1099800420941606 32691605 PMC8822203

[fsn33538-bib-0067] McDermott, M. T. , & Ridgway, E. C. (2001). Diagnosis and treatment of hypothyroidism. In Practical management of thyroid disorders (pp. 135–186). Marcel Dekker, Inc.

[fsn33538-bib-0068] Milanesi, A. , & Brent, G. A. (2017). Chapter 12 – Iodine and thyroid hormone synthesis, metabolism, and action. In J. F. Collins (Ed.), Molecular, genetic, and nutritional aspects of major and trace minerals (pp. 143–150). Academic Press.

[fsn33538-bib-0069] Moline, J. , & Eng, C. (2011). Multiple endocrine neoplasia type 2: An overview. Genetics in Medicine, 13(9), 755–764. 10.1097/GIM.0b013e318216cc6d 21552134

[fsn33538-bib-0070] Mouhamed, D. H. , Ezzaher, A. , Neffati, F. , Douki, W. , Gaha, L. , & Najjar, M. F. (2012). Risk of hyperthyroidism in a Tunisian population of smokers. Annales de Biologie Clinique, 70(2), 199–206. 10.1684/abc.2012.0689 22484531

[fsn33538-bib-0071] Naoum, G. E. , Morkos, M. , Kim, B. , & Arafat, W. (2018). Novel targeted therapies and immunotherapy for advanced thyroid cancers. Molecular Cancer, 17(1), 51. 10.1186/s12943-018-0786-0 29455653 PMC5817719

[fsn33538-bib-0072] Oliveira, K. J. , Chiamolera, M. I. , Giannocco, G. , Pazos‐Moura, C. C. , & Ortiga‐Carvalho, T. M. (2018). Thyroid function disruptors: From nature to chemicals. Journal of Molecular Endocrinology, 62, R1–R19. 10.1530/jme-18-0081 30006341

[fsn33538-bib-0073] Otun, J. , Sahebkar, A. , Östlundh, L. , & Atkin, S. L. (2019). Systematic review and meta‐analysis on the effect of soy on thyroid function. Scientific Reports, 9(1), 3964. 10.1038/s41598-019-40647-x 30850697 PMC6408586

[fsn33538-bib-0074] Owen, D. H. , Konda, B. , Sipos, J. , Liu, T. , Webb, A. , Ringel, M. D. , Timmers, C. D. , & Shah, M. H. (2019). KRAS G12V mutation in acquired resistance to combined BRAF and MEK inhibition in papillary thyroid cancer. Journal of the National Comprehensive Cancer Network, 17(5), 409–413. 10.6004/jnccn.2019.7292 31085763 PMC6673655

[fsn33538-bib-0075] Pace‐Asciak, P. , Russell, J. O. , & Tufano, R. P. (2022). Surgical treatment of thyroid cancer: Established and novel approaches. Best Practice & Research Clinical Endocrinology & Metabolism, 37, 101664. 10.1016/j.beem.2022.101664 35534363

[fsn33538-bib-0122] Parker, D. R. (2009). Perchlorate in the environment: the emerging emphasis on natural occurrence. Environmental Chemistry, 6, 10–27. 10.1071/EN09001

[fsn33538-bib-0076] Paul, R. F. , Hassan, M. , Nazar, H. S. , Gillani, S. , Afzal, N. , & Qayyum, I. (2011). Effect of body mass index on serum leptin levels. Journal of Ayub Medical College, Abbottabad, 23(3), 40–43. 23272432

[fsn33538-bib-0077] Punatar, S. B. , Noronha, V. , Joshi, A. , & Prabhash, K. (2012). Thyroid cancer in Gardner's syndrome: Case report and review of literature. South Asian Journal of Cancer, 1(1), 43–47. 10.4103/2278-330x.96510 24455508 PMC3876602

[fsn33538-bib-0078] Rehman, H. , Böhm, J. , & Zentek, J. (2008). Effects of differentially fermentable carbohydrates on the microbial fermentation profile of the gastrointestinal tract of broilers. Journal of Animal Physiology and Animal Nutrition, 92(4), 471–480. 10.1111/j.1439-0396.2007.00736.x 18662357

[fsn33538-bib-0121] Rickard, B. P. , Rizvi, I. , & Fenton, S. E. (2022). Per‐ and poly‐fluoroalkyl substances (PFAS) and female reproductive outcomes: PFAS elimination, endocrine‐mediated effects, and disease. Toxicology, 465. 10.1016/j.tox.2021.153031 PMC8743032 34774661

[fsn33538-bib-0079] Rinninella, E. , Raoul, P. , & Cintoni, M. (2019). What is the healthy gut microbiota composition? A changing ecosystem across age, environment, diet, and diseases. Microorganisms, 7(1), 14. 10.3390/microorganisms7010014 30634578 PMC6351938

[fsn33538-bib-0080] Roman, G. C. (2007). Autism: Transient in utero hypothyroxinemia related to maternal flavonoid ingestion during pregnancy and to other environmental antithyroid agents. Journal of the Neurological Sciences, 262(1–2), 15–26. 10.1016/j.jns.2007.06.023 17651757

[fsn33538-bib-0081] Russell, J. O. , & Razavi, C. R. (2021). Transoral thyroidectomy: Safety and outcomes of 200 consecutive North American cases. World Journal of Surgery, 45(3), 774–781. 10.1007/s00268-020-05874-8 33205227

[fsn33538-bib-0082] Santoro, M. , Moccia, M. , Federico, G. , & Carlomagno, F. (2020). RET gene fusions in malignancies of the thyroid and other tissues. Genes, 11(4), 424. 10.3390/genes11040424 32326537 PMC7230609

[fsn33538-bib-0083] Sanyal, D. , & Raychaudhuri, M. (2016). Hypothyroidism and obesity: An intriguing link. Indian Journal of Endocrinology and Metabolism, 20(4), 554–557. 10.4103/2230-8210.183454 27366725 PMC4911848

[fsn33538-bib-0084] Schomburg, L. (2012). Selenium, selenoproteins and the thyroid gland: Interactions in health and disease. Nature Reviews Endocrinology, 8(3), 160–171. 10.1038/nrendo.2011.174 22009156

[fsn33538-bib-0085] Shah, M. D. , Hall, F. T. , Eski, S. J. , Witterick, I. J. , Walfish, P. G. , & Freeman, J. L. (2003). Clinical course of thyroid carcinoma after neck dissection. Laryngoscope, 113(12), 2102–2107. 10.1097/00005537-200312000-00008 14660910

[fsn33538-bib-0086] Shen, F. , Cai, W. S. , Li, J. L. , Feng, Z. , Cao, J. , & Xu, B. (2015). The association between serum levels of selenium, copper, and magnesium with thyroid cancer: A meta‐analysis. Biological Trace Element Research, 167(2), 225–235. 10.1007/s12011-015-0304-9 25820485

[fsn33538-bib-0087] Shrestha, G. , Chang, C.‐P. , Pun, C. B. , Gautam, D. K. , Siwakoti, B. , Sapkota, A. , & Hashibe, M. (2023). Differences in risk factors for head and neck cancer among men and women in Nepal: A case‐control study. Cancer Epidemiology, 82, 102319. 10.1016/j.canep.2022.102319 36566578 PMC9852028

[fsn33538-bib-0088] Sohail, M. U. , Ijaz, A. , Yousaf, M. S. , Ashraf, K. , Zaneb, H. , Aleem, M. , & Rehman, H. (2010). Alleviation of cyclic heat stress in broilers by dietary supplementation of mannan‐oligosaccharide and *Lactobacillus*‐based probiotic: Dynamics of cortisol, thyroid hormones, cholesterol, C‐reactive protein, and humoral immunity. Poultry Science, 89(9), 1934–1938. 10.3382/ps.2010-00751 20709978

[fsn33538-bib-0089] Song, Q. , Chen, X. , Su, Y. , Xie, Z. , Wang, S. , & Cui, B. (2019). Age and gender specific thyroid hormones and their relationships with body mass index in a large Chinese population. International Journal of Endocrinology and Metabolism, 17(1), e66450. 10.5812/ijem.66450 30881465 PMC6408740

[fsn33538-bib-0090] Su, X. , Li, M. , Liu, L. , Shen, H. , Kelly, P. J. , Wang, Y. , Chen, Z. , Wang, J. , Li, W. , Chen, H. , Xiao, B. , Han, Y. , Liu, S. , & Liu, P. (2018). Assessment of thyroid function in children, adults and pregnant and lactating women after long‐term salt iodisation measurements. British Journal of Nutrition, 119(11), 1245–1253. 10.1017/s0007114518000570 29580306

[fsn33538-bib-0091] Su, X. , Zhao, Y. , Li, Y. , Ma, S. , & Wang, Z. (2020). Gut dysbiosis is associated with primary hypothyroidism with interaction on gut‐thyroid axis. Clinical Science, 134(12), 1521–1535. 10.1042/cs20200475 32519746

[fsn33538-bib-0092] Subbiah, V. , Baik, C. , & Kirkwood, J. M. (2020). Clinical development of BRAF plus MEK inhibitor combinations. Trends in Cancer, 6(9), 797–810. 10.1016/j.trecan.2020.05.009 32540454

[fsn33538-bib-0093] Suzuki, K. , Iwai, H. , Utsunomiya, K. , Kono, Y. , Kobayashi, Y. , Van Bui, D. , Sawada, S. , Yun, Y. , Mitani, A. , Kondo, N. , Katano, T. , Tanigawa, N. , Akama, T. , & Kanda, A. (2021). Combination therapy with lenvatinib and radiation significantly inhibits thyroid cancer growth by uptake of tyrosine kinase inhibitor. Experimental Cell Research, 398(1), 112390. 10.1016/j.yexcr.2020.112390 33227314

[fsn33538-bib-0094] Taylor, P. N. , Richmond, R. , Davies, N. , Sayers, A. , Stevenson, K. , Woltersdorf, W. , Taylor, A. , Groom, A. , Northstone, K. , Ring, S. , Okosieme, O. , Rees, A. , Nitsch, D. , Williams, G. R. , Smith, G. D. , Gregory, J. W. , Timpson, N. J. , Tobias, J. H. , & Dayan, C. M. (2016). Paradoxical relationship between body mass index and thyroid hormone levels: A study using Mendelian randomization. The Journal of Clinical Endocrinology and Metabolism, 101(2), 730–738. 10.1210/jc.2015-3505 26595101 PMC4880123

[fsn33538-bib-0095] Teng, Y. , Ren, C. , Chen, X. , Shen, Y. , Zhang, Z. , Chai, L. , & Wang, H. (2022). Effects of cadmium exposure on thyroid gland and endochondral ossification in *Rana zhenhaiensis* . Environmental Toxicology and Pharmacology, 92, 103860. 10.1016/j.etap.2022.103860 35367624

[fsn33538-bib-0096] Terezakis, S. A. , & Lee, N. Y. (2010). The role of radiation therapy in the treatment of medullary thyroid cancer. Journal of the National Comprehensive Cancer Network, 8(5), 532–540; quiz 541. 10.6004/jnccn.2010.0041 20495083

[fsn33538-bib-0097] Tian, T. , Huang, R. , & Liu, B. (2019). Is TSH suppression still necessary in intermediate‐ and high‐risk papillary thyroid cancer patients with pre‐ablation stimulated thyroglobulin <1 ng/mL before the first disease assessment? Endocrine, 65(1), 149–154. 10.1007/s12020-019-01914-z 30924085

[fsn33538-bib-0098] Trapani, K. M. , Boghossian, L. J. , & Caskey, E. (2018). *Clostridium subterminale* septicemia in a patient with metastatic gastrointestinal adenocarcinoma. Case Reports in Infectious Diseases, 2018, 1–3. 10.1155/2018/6031510 PMC5987309 29951328

[fsn33538-bib-0099] Triggiani, V. , Tafaro, E. , Giagulli, V. A. , Sabba, C. , Resta, F. , Licchelli, B. , & Guastamacchia, E. (2009). Role of iodine, selenium and other micronutrients in thyroid function and disorders. Endocrine, Metabolic & Immune Disorders – Drug Targets, 9(3), 277–294. 10.2174/187153009789044392 19594417

[fsn33538-bib-0100] Tuominen, H. , & Rautava, J. (2021). Oral microbiota and cancer development. Pathobiology, 88(2), 116–126. 10.1159/000510979 33176328

[fsn33538-bib-0101] Valeix, P. , Faure, P. , Bertrais, S. , Vergnaud, A. C. , Dauchet, L. , & Hercberg, S. (2008). Effects of light to moderate alcohol consumption on thyroid volume and thyroid function. Clinical Endocrinology, 68(6), 988–995. 10.1111/j.1365-2265.2007.03123.x 18031329

[fsn33538-bib-0102] Valerio, L. , Maino, F. , Castagna, M. G. , & Pacini, F. (2022). Radioiodine therapy in the different stages of differentiated thyroid cancer. Best Practice & Research Clinical Endocrinology & Metabolism, 37, 101703. 10.1016/j.beem.2022.101703 36151009

[fsn33538-bib-0103] Vashishta, R. , Mahalingam‐Dhingra, A. , Lander, L. , Shin, E. J. , & Shah, R. K. (2012). Thyroidectomy outcomes: A national perspective. Otolaryngology and Head and Neck Surgery, 147(6), 1027–1034. 10.1177/0194599812454401 22807486

[fsn33538-bib-0104] Vigneri, R. , Malandrino, P. , Gianì, F. , Russo, M. , & Vigneri, P. (2017). Heavy metals in the volcanic environment and thyroid cancer. Molecular and Cellular Endocrinology, 457, 73–80. 10.1016/j.mce.2016.10.027 27794445

[fsn33538-bib-0105] Virili, C. , Fallahi, P. , Antonelli, A. , Benvenga, S. , & Centanni, M. (2018). Gut microbiota and Hashimoto's thyroiditis. Reviews in Endocrine and Metabolic Disorders, 19(4), 293–300. 10.1007/s11154-018-9467-y 30294759

[fsn33538-bib-0125] Virili, C. , Stramazzo, I. , & Centanni, M. (2021). Gut microbiome and thyroid autoimmunity. Best Practice & Research Clinical Endocrinology & Metabolism, 35. 10.1016/j.beem.2021.101506 33648848

[fsn33538-bib-0106] Werner, S. C. , Ingbar, S. H. , Braverman, L. E. , & Utiger, R. D. (2005). Werner & Ingbar's the thyroid: A fundamental and clinical text (Vol. 549). Lippincott Williams & Wilkins.

[fsn33538-bib-0107] Wiersinga, W. M. (2013). Smoking and thyroid. Clinical Endocrinology, 79(2), 145–151. 10.1111/cen.12222 23581474

[fsn33538-bib-0108] Winther, K. H. , Rayman, M. P. , Bonnema, S. J. , & Hegedus, L. (2020). Selenium in thyroid disorders – Essential knowledge for clinicians. Nature Reviews Endocrinology, 16(3), 165–176. 10.1038/s41574-019-0311-6 32001830

[fsn33538-bib-0109] Xu, M. , Zheng, Y. , Zuo, Z. , Zhou, Q. , Deng, Q. , Wang, J. , & Wang, D. (2023). De novo familial adenomatous polyposis associated thyroid cancer with a c.2929delG frameshift deletion mutation in APC: A case report and literature review. World Journal of Surgical Oncology, 21(1), 73. 10.1186/s12957-023-02951-9 36864485 PMC9979514

[fsn33538-bib-0110] Xu, R. , Huang, F. , Zhang, S. , Lv, Y. , & Liu, Q. (2019). Thyroid function, body mass index, and metabolic risk markers in euthyroid adults: A cohort study. BMC Endocrine Disorders, 19(1), 58. 10.1186/s12902-019-0383-2 31174521 PMC6555987

[fsn33538-bib-0111] Yao, R. , Chiu, C. G. , Strugnell, S. S. , Gill, S. , & Wiseman, S. M. (2011). Gender differences in thyroid cancer: A critical review. Expert Review of Endocrinology and Metabolism, 6(2), 215–243. 10.1586/eem.11.9 30290447

[fsn33538-bib-0112] Yasmin, A. , Butt, M. S. , Afzaal, M. , van Baak, M. , Nadeem, M. T. , & Shahid, M. Z. (2015). Prebiotics, gut microbiota and metabolic risks: Unveiling the relationship. Journal of Functional Foods, 17, 189–201. 10.1016/j.jff.2015.05.004

[fsn33538-bib-0113] Yu, X. , Jiang, W. , Kosik, R. O. , Song, Y. , Luo, Q. , Qiao, T. , Tong, J. , Liu, S. , Deng, C. , Qin, S. , Lv, Z. , & Li, D. (2022). Gut microbiota changes and its potential relations with thyroid carcinoma. Journal of Advanced Research, 35, 61–70. 10.1016/j.jare.2021.04.001 35003794 PMC8721249

[fsn33538-bib-0114] Yuan, L. , Yang, P. , Wei, G. , Hu, X. , Chen, S. , Lu, J. , Yang, L. , He, X. , & Bao, G. (2022). Tumor microbiome diversity influences papillary thyroid cancer invasion. Communications Biology, 5(1), 864. 10.1038/s42003-022-03814-x 36002642 PMC9402670

[fsn33538-bib-0115] Zhang, J. , Zhang, F. , Zhao, C. , Xu, Q. , Liang, C. , Yang, Y. , Wang, H. , Shang, Y. , Wang, Y. , Mu, X. , Zhu, D. , Zhang, C. , Yang, J. , Yao, M. , & Zhang, L. (2019). Dysbiosis of the gut microbiome is associated with thyroid cancer and thyroid nodules and correlated with clinical index of thyroid function. Endocrine, 64(3), 564–574. 10.1007/s12020-018-1831-x 30584647

[fsn33538-bib-0116] Zhang, Y. , Shi, L. , Zhang, Q. , Peng, N. , Chen, L. , Lian, X. , Liu, C. , Shan, Z. , Shi, B. , Tong, N. , Wang, S. , Weng, J. , Zhao, J. , & Teng, W. (2019). The association between cigarette smoking and serum thyroid stimulating hormone, thyroid peroxidase antibodies and thyroglobulin antibodies levels in Chinese residents: A cross‐sectional study in 10 cities. PLoS One, 14(11), e0225435. 10.1371/journal.pone.0225435 31765419 PMC6876836

[fsn33538-bib-0124] Zhao, F. , Feng, J. , Li, J. , Zhao, L. , Liu, Y. , Chen, H. , Jin, Y. , Zhu, B. , & Wei, Y. (2018). Alterations of the Gut Microbiota in Hashimoto's Thyroiditis Patients. Thyroid, 28, 175–186. 10.1089/thy.2017.0395 29320965

[fsn33538-bib-0117] Zhao, Y. , Zhang, J. , Han, X. , & Fan, S. (2019). Total body irradiation induced mouse small intestine senescence as a late effect. Journal of Radiation Research, 60(4), 442–450. 10.1093/jrr/rrz026 31165161 PMC6641339

[fsn33538-bib-0118] Zheng, L. , Zhang, L. , Tang, L. , Huang, D. , Pan, D. , Guo, W. , He, S. , Huang, Y. , Chen, Y. , Xiao, X. , Tang, B. , & Chen, J. (2023). Gut microbiota is associated with response to 131I therapy in patients with papillary thyroid carcinoma. European Journal of Nuclear Medicine and Molecular Imaging, 50(5), 1453–1465. 10.1007/s00259-022-06072-5 36512067 PMC10027784

[fsn33538-bib-0119] Zimmermann, M. B. , & Boelaert, K. (2015). Iodine deficiency and thyroid disorders. The Lancet Diabetes and Endocrinology, 3(4), 286–295. 10.1016/s2213-8587(14)70225-6 25591468

